# 2021 ISHNE/ HRS/ EHRA/ APHRS collaborative statement on mHealth in Arrhythmia Management: Digital Medical Tools for Heart Rhythm Professionals: From the International Society for Holter and Noninvasive Electrocardiology/Heart Rhythm Society/European Heart Rhythm Association/Asia Pacific Heart Rhythm Society

**DOI:** 10.1111/anec.12795

**Published:** 2021-01-29

**Authors:** Niraj Varma, Iwona Cygankiewicz, Mintu Turakhia, Hein Heidbuchel, Yufeng Hu, Lin Yee Chen, Jean‐Philippe Couderc, Edmond M. Cronin, Jerry D. Estep, Lars Grieten, Deirdre A. Lane, Reena Mehra, Alex Page, Rod Passman, Jonathan Piccini, Ewa Piotrowicz, Ryszard Piotrowicz, Pyotr G. Platonov, Antonio Luiz Ribeiro, Robert E. Rich, Andrea M. Russo, David Slotwiner, Jonathan S. Steinberg, Emma Svennberg

**Affiliations:** ^1^ Cleveland Clinic Cleveland OH USA; ^2^ Medical University of Lodz Lodz Poland; ^3^ Stanford University Palo Alto CA USA; ^4^ Antwerp University and University Hospital Antwerp Belgium; ^5^ Taipei Veterans General Hospital Taipei Taiwan; ^6^ University of Minnesota Minneapolis MN USA; ^7^ University of Rochester Rochester NY USA; ^8^ Temple University Philadelphia PA USA; ^9^ Hasselt University Hasselt Belgium; ^10^ University of Liverpool Liverpool UK; ^11^ Northwestern University Feinberg School of Medicine Chicago IL USA; ^12^ Duke University Durham NC USA; ^13^ National Institute of Cardiology Warsaw Poland; ^14^ Lund University Lund Sweden; ^15^ Faculdade de Medicina Centro de Telessaúde, Hospital das Clínicas, and Departamento de Clínica Médica Universidade Federal de Minas Gerais Belo Horizonte Brazil; ^16^ Cooper Medical School of Rowan University Camden NJ USA; ^17^ Cardiology Division NewYork‐Presbyterian Queens, and School of Health Policy and Research Weill Cornell Medicine New York NY USA; ^18^ Karolinska University Hospital Stockholm Sweden

**Keywords:** arrhythmias, digital medicine, heart rhythm, atrial fibrillation, comorbidities, mHealth

## Abstract

This collaborative statement from the International Society for Holter and Noninvasive Electrocardiology/ Heart Rhythm Society/ European Heart Rhythm Association/ Asia Pacific Heart Rhythm Society describes the current status of mobile health ("mHealth") technologies in arrhythmia management. The range of digital medical tools and heart rhythm disorders that they may be applied to and clinical decisions that may be enabled are discussed. The facilitation of comorbidity and lifestyle management (increasingly recognized to play a role in heart rhythm disorders) and patient self‐management are novel aspects of mHealth. The promises of predictive analytics but also operational challenges in embedding mHealth into routine clinical care are explored.

**Abbreviations**
AI: artificial intelligence
ACC: American College of Cardiology
ACS: acute coronary syndrome
AED: automated external defibrillator
AF: atrial fibrillation
AHA: American Heart Association
AHRE: atrial high‐rate episode
APHRS: Asia Pacific Heart Rhythm Society
BP: blood pressure
CIED: cardiovascular implantable electronic device
CPR: cardiopulmonary resuscitation
EHRA: European Heart Rhythm Association
EMR: electronic medical record
ESUS: embolic stroke of unknown source
FDA (U.S.): Food and Drug Administration
GPS: global positioning system
HCP: healthcare professional
HF: heart failure
HR: heart rate
HRS: Heart Rhythm Society
ICD: implantable cardioverter‐defibrillator
ILR: implantable loop recorder
ISHNE: International Society for Holter and Noninvasive Electrocardiology
JITAI: just‐in‐time adaptive intervention
MCT: mobile cardiac telemetry
OAC: oral anticoagulant
PAC: premature atrial complex
PPG: photoplethysmography
PVC: premature ventricular complexes
SCA: sudden cardiac arrest
TADA: Technology Assissted Dietary Assessment
VT: ventricular tachycardia

**Table:** 

1. INTRODUCTION	3
Document scope and rationale	3
2. mHEALTH TECHNOLOGIES	6
2.1. Ambulatory ECG monitoring	6
2.2. New mHealth‐based modalities for arrhythmia monitoring	7
2.2.1 ECG‐based	9
2.2.1.1. Handheld devices	9
2.2.1.2. Wearable patches	9
2.2.1.3. Biotextiles	9
2.2.1.4. Smartphone and smartwatch‐based devices	10
2.2.2 Non‐ECG‐based	11
2.2.2.1. Photoplethysmography	11
2.2.2.2. Oscillometry	12
2.2.2.3. Mechanocardiography	12
2.2.2.4. Contactless video plethysmography	12
2.2.2.5. Smart speakers	12
3. mHEALTH APPLICATIONS FOR ARRHYTHMIAS	15
3.1. Atrial fibrillation	15
3.1.1. Undiagnosed atrial fibrillation identification	17
3.1.2. Targeted identification in high‐risk individuals	18
3.1.3. Diagnostics in people with established atrial fibrillation	19
3.1.4. Atrial fibrillation therapy	19
3.2. Sudden cardiac death	20
4. COMORBIDITIES	25
4.1. Ischemic heart disease	26
4.2. Heart failure	27
4.2.1. Mobile technologies for managing heart failure	28
4.2.2. Hybrid telerehabilitation in patients with heart failure	28
4.3. Diabetes	29
4.4. Hypertension	29
4.5. Disorders including sleep apnea	30
4.6. Lifestyle	30
4.6.1. Physical activity	30
4.6.2. Diet	31
5. PATIENT SELF‐MANAGEMENT—INTEGRATED CHRONIC CARE	36
5.1. Patient engagement	36
5.2. Behavioral modification	37
5.3. Patients as part of a community	37
5.4. Maintaining patient engagement	37
5.5. Digital divide	38
6. CLINICAL TRIALS	41
7. OPERATIONAL CHALLENGES	43
7.1. Healthcare system—eHealth monitoring and hospital ecosystem	43
7.2. Cybersecurity guidance for mHealth devices	44
7.2.1. Hacking strategies and methods in mHealth technologies	44
7.2.2. Recommendations to the manufacturer	45
7.2.3. Recommendations to clinicians and administrator	45
7.2.4. Recommendations to patients	45
7.3. Reimbursement	45
7.4. Regulatory landscape for mHealth devices	46
8. Predictive analytics	48
9. Future directions	49

## Central Figure

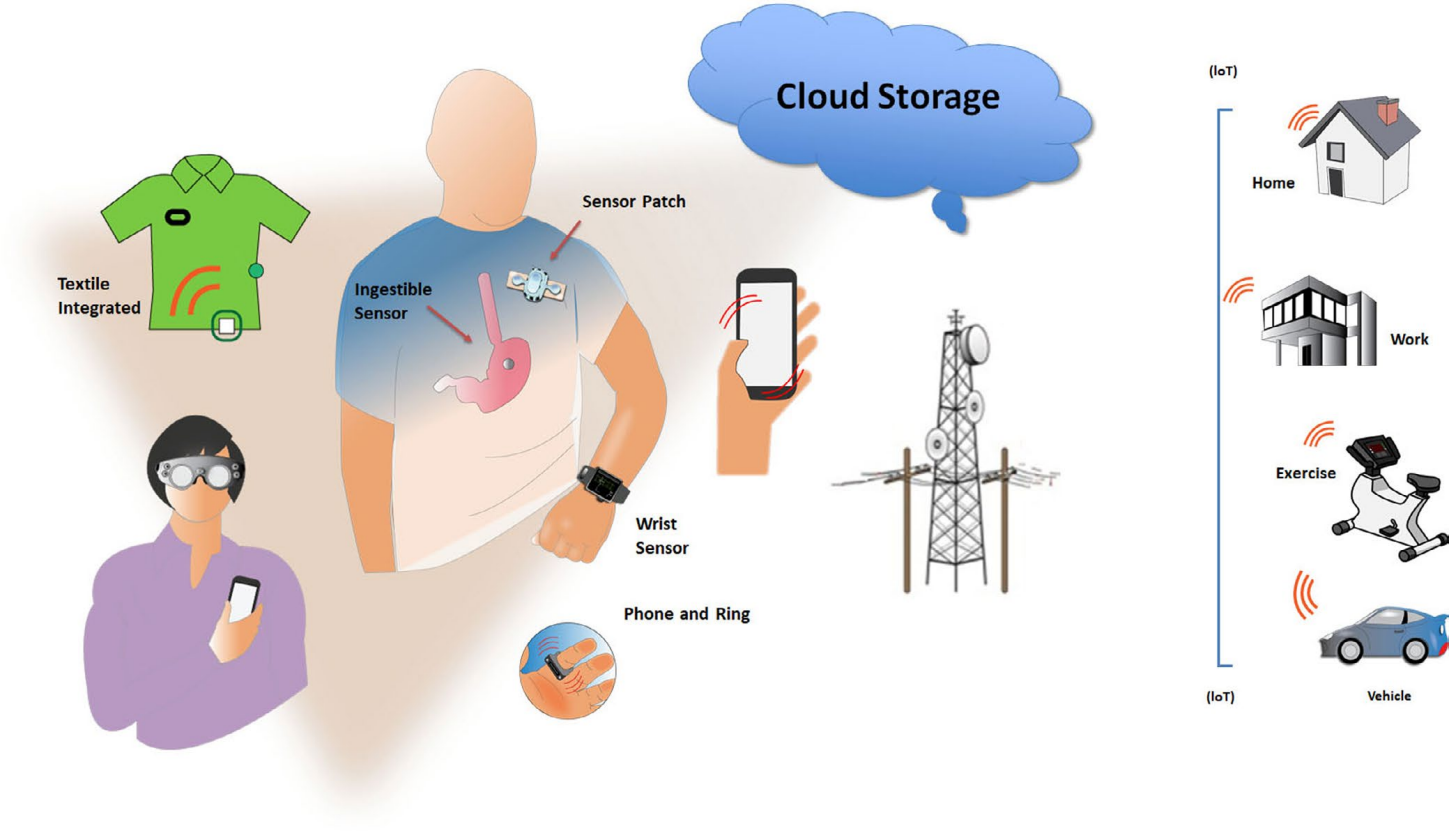

**FIGURE LEGEND:** mHealth tools for the individual. Sensors can be embedded in a variety of wearables. (IoT: Internet of things—connects from any location to hospital or cloud; See Table 1).

**Table 1:** mHealth‐based modalities for arrhythmia monitoring

	Signal acquisition and visualization	ECG duration	Signal storage and transmission	Indications/Populations tested	Advantages	Limitations
ECG‐based devices						
Handheld	External sensors; Single or multilead ECG on demand; Display in‐screen ECG or screen of PC/laptop/smartphone, after transmission or real‐time ECG analysis available	Intermittent Recording: 10 sec to 2 min	Built‐in memory Bluetooth WiFi	Palpitations AF screening	Easy to use Low cost	Short ECG duration
Wearable patches	Built‐in electrodes Patch attached to the skin	Continuous recording Up to 14 days	Built‐in memory with post hoc analysis, or Bluetooth transmission with real‐time analysis in selected devices	Low‐risk patients with palpitations and syncope; AF screening	Continuous longer‐term ECG recording; Built‐in alarm button High patients’ compliance; Patients can affix at home Water‐resistant	Single‐channel ECG Skin irritation
Biotextiles	Electrodes/sensors embedded into biotextile—vests, belts Single or multichannel	Continuous recording up to 30 days	Built‐in memory Real‐time Bluetooth transmission	Low‐risk patients with palpitations and syncope AF screening	Continuous long‐term recording; Built‐in alarm button; High patients’ acceptance and adherence; Multiparameter evaluation; Can be used as monitoring and treating device (WCD)	Limited Availability Movement artifacts
Smartphone‐based	External sensors attached to mobile phone Single/ multilead ECG Real‐time ECG on smartphone’s screen or PC/laptop after transmission	Intermittent recording up to 30 sec Patient activated	Built‐in memory Real‐time or post hoc transmission	Low‐risk patients with palpitations AF screening	Widely available Long‐life possibility of intermittent recording	Intermittent recording
Smartwatch‐based	Built‐in sensors	Intermittent recording Patient activated	Built‐in memory Real‐time or post hoc transmission	Low‐risk patients with palpitations AF screening	Widely available Long‐life possibility of intermittent recording	Intermittent recording Single‐channel
*Non‐ECG‐based*						
Photoplethysmography (PPG)	HR from changes in reflectance of the tissue blood volume of a skin surface	Intermittent patient activated in smartphones Continuous measurement of HR in smartwatches and wristbands	Built‐in memory Real‐time or post hoc transmission	Low‐risk patients with palpitations AF screening HR measurement during physical activity	Widely available	Irregular heart‐presumed AF
Oscillometry	BP monitors with HR measurement	Intermittent recording during BP measurement	Built‐in memory Post hoc transmission	HR assessment Opportunistic AF screening	Widely available	Irregular heart‐presumed AF
Video recording	Camera from smartphones, TVs	Patient activated Continuous recording in prespecified time frame	Real‐time or post hoc transmission	Low‐risk patients with palpitations AF screening Undiagnosed falls	Can use existing cameras from household goods	Irregular heart‐presumed AF Limited availability

Abbreviations: AF, atrial fibrillation; BP, blood pressure; HR, heart rate; WCD, wearable cardioverter‐defibrillator.

## 1 INTRODUCTION

### Document scope and rationale

Digital health is an umbrella term to describe the use of digital information, data, and communication technologies to collect, share, and analyze health information in order to improve patient health, education, and healthcare delivery (https://www.fcc.gov/general/five‐questions‐you‐can‐ask‐your‐doctor‐about‐digital‐health#ab) (*Turakhia, Desai, & Harrington, 2016*). This concept encompasses telehealth, electronic health records, implantable device monitoring, wearable sensor data, analytics and artificial intelligence (AI), behavioral health, and personalized medicine. Among these, mobile health—or “mHealth” is a component of digital health, defined by the World Health Organization—as “medical and public health practice supported by mobile devices, such as mobile phones, patient monitoring devices, personal digital assistants (PDAs), and other wireless devices” (https://www.who.int/goe/publications/goe_mhealth_web.pdf) (https://apps.who.int/gb/ebwha/pdf_files/WHA71/A71_20‐en.pdf?ua=1). Utilization of these devices has proliferated among health‐conscious consumers in recent years and is likely to continue rapid expansion and integration into more formalized medical settings.

mHealth flows intuitively to health professionals in the field of arrhythmia management from experience gained through remote monitoring of cardiovascular implantable electronic devices (CIEDs), such as pacemakers and implantable cardioverter‐defibrillators (ICDs) (*Varma, Epstein, Irimpen, Schweikert, & Love, 2010*). A wealth of data garnered from many studies over the last 10‐15 years have confirmed the benefits of remote technology‐assisted follow‐up and established it as standard of care (*Varma & Ricci, 2013; Slotwiner et al., 2019*). However, results of remote monitoring of CIEDs may not be immediately generalizable to mHealth. For instance, the former is restricted to those with cardiac disease (largely arrhythmias and heart failure (HF)), that is, a group already defined as patients. The care pathways for CIED remote monitoring are also well defined, with billing and reimbursement in place in the United States and many other parts of the world. In comparison, mHealth differs: It is widely available in the form of consumer products that penetrate most sectors of society, including individuals without formal medical diagnoses; it may be applied to a wider group of medical conditions; data can be self‐monitored rather than assessed by healthcare professionals (HCPs); and reimbursement models are not mature. Indeed, some heart rhythm tracking capabilities may be indirectly acquired in products purchased for different goals and then subsequently used for self‐monitoring. Conversely, in the medical space, applications are largely not prescribed by HCPs, often lack validation for disease management use cases, and care pathways remain varied or poorly defined. Nevertheless, if properly implemented, the intersection of these two communities opens up a broad spectrum of opportunities, extending from population screening and surveillance for undiagnosed disease to longitudinal disease management, and importantly, engaging patients in their own cycle of care, allowing much health care to be asynchronous and virtualized. Its value and degree of integration will depend on different healthcare systems in different countries (Table 1).

mHealth has value only if the acquired information leads to decisions that improve outcome. This requires a clear path of information flow and actionability. Moreover, all stakeholders need to be aware of the logistical chain (so that everyone knows what to expect) and responsibilities clearly defined (possibly including device vendors). Similarly, actions taken based on the monitored information should be transparent to all stakeholders. For example, for a patient who records and transmits an irregular heart rhythm via a wearable device, a designated decision process should be followed to confirm eg whether the rhythm is atrial fibrillation (AF) or not, whether confirmation by another diagnostic test is required, how that is arranged, and finally what therapy should be implemented and in what reasonable time frame? Clearly, there are risks of increasing cost from medical testing and provoking anxiety in consumers—who by virtue of seeking a medical verification become patients. Again, CIED experience sets a precedent. Studies that have shown improved outcome with telemonitoring succeeded when integrated into a clear logistical framework for a specific use case of disease management (e.g., IN‐TIME for remote monitoring in patients receiving cardiac resynchronization therapy, CardioMEMS) (*Abraham et al., 2011*, *Hindricks et al., 2014*, *Varma & Ricci, 2013*). Replicating this with mHealth creates challenges for healthcare providers and goes far beyond the technological capabilities of the monitoring and transmission equipment. Implementation will require defined aims and fundamental changes to existing workflows and responsibilities. Such changes are always difficult. Apart from the organizational issues required to achieve such changes, reimbursement may drive or hinder such changes in the workplace. Awareness of these factors has been heightened by the SARS‐CoV‐2 pandemic, during which telemedicine solutions have been advocated to reduce patient contact with healthcare providers yet continue healthcare delivery (*Varma et al., 2020*).

In view of the rapid technological development and popularity of wearable and other mobile devices, and the need for analysis and planning of the mHealth infrastructure, ISHNE (International Society for Holter and Noninvasive Electrocardiology), HRS (Heart Rhythm Society), EHRA (European Heart Rhythm Association), and APHRS (Asia Pacific Heart Rhythm Society), recognized the need for this collaborative statement. The aim of this document is to define state‐of‐the‐art mHealth technologies and their application in arrhythmia management and explore future directions for clinical application. As such, the scope of the document encompasses discussion of the different mHealth technologies currently available or in development; the acquisition of health‐related data; the applications of such data, including disease identification and management; clinical trials; the patient perspective; and the issues that must be addressed in the future to permit useful application of mHealth technologies. Addtionally, discussion is extended to mHealth facilitation of those comorbidities increasingly recognized to influence arrhythmia management (e.g., obesity and sleep apnea) that are becoming the responsibility of heart rhythm professionals (*Chung et al., 2020*).

## 2 mHEALTH TECHNOLOGIES

**Figure 1:**
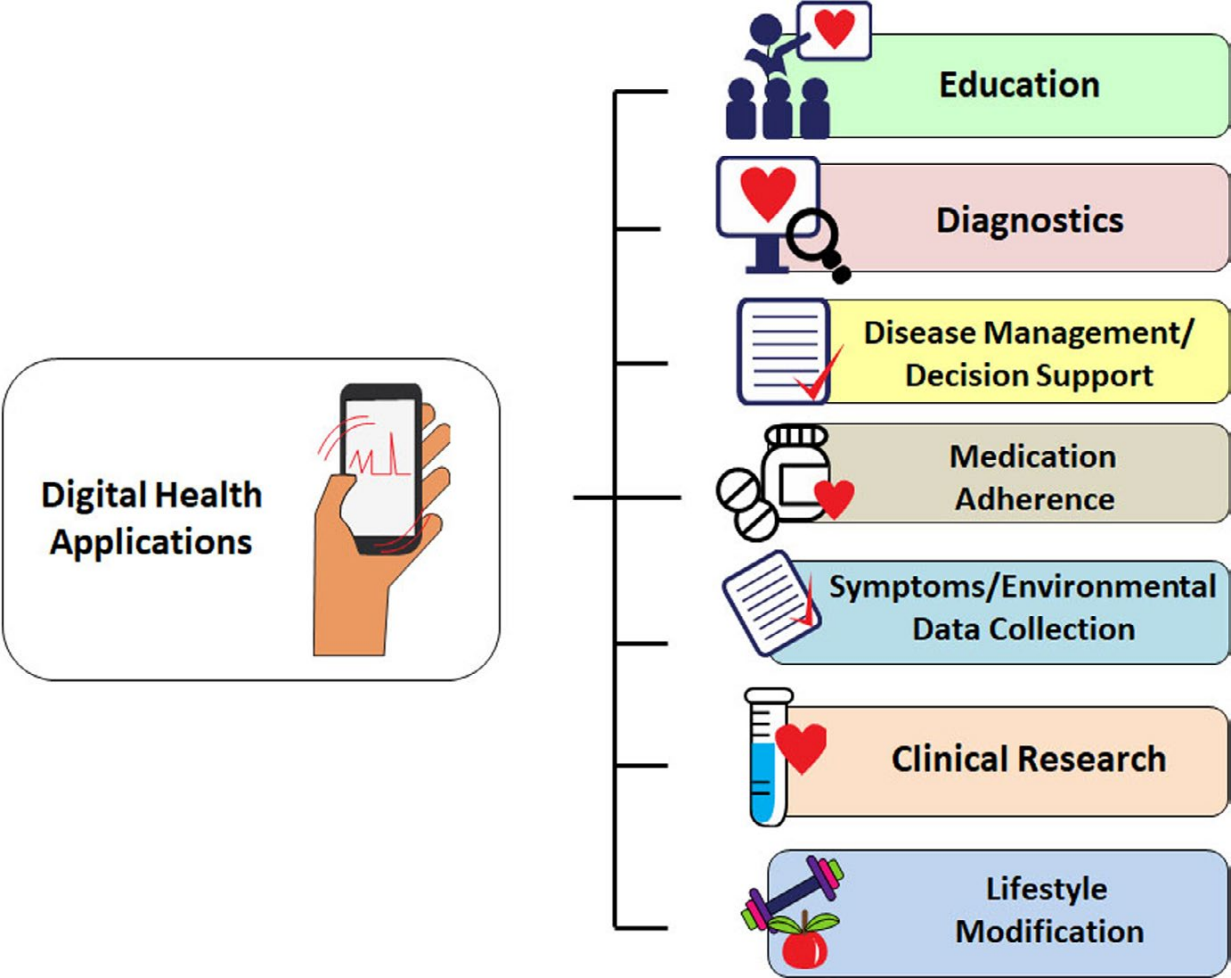
Application of digital health technologies in arrhythmias (Many of these sectors are interconnected).

Dedicated applications and sensors, within or adjunctive to mobile communication devices, enable users to monitor, collect, and share physiologic and health data. Their applications range from diagnostic, decision support, disease management, evaluation of medication adherence, and for educational and clinical research purposes (Figure 1). They synergize naturally with arrhythmia evaluation and extend management to associated comorbidities and lifestyle.

Applications to arrhythmias

- Diagnostic
  - Evaluate patients with symptoms suggestive of arrhythmias
  - Assess patients’ response to both pharmacological and invasive treatment of arrhythmias.
- Screening
  - Increasing emphasis on AF.

### 2.1 Ambulatory ECG monitoring

**Figure 2:**
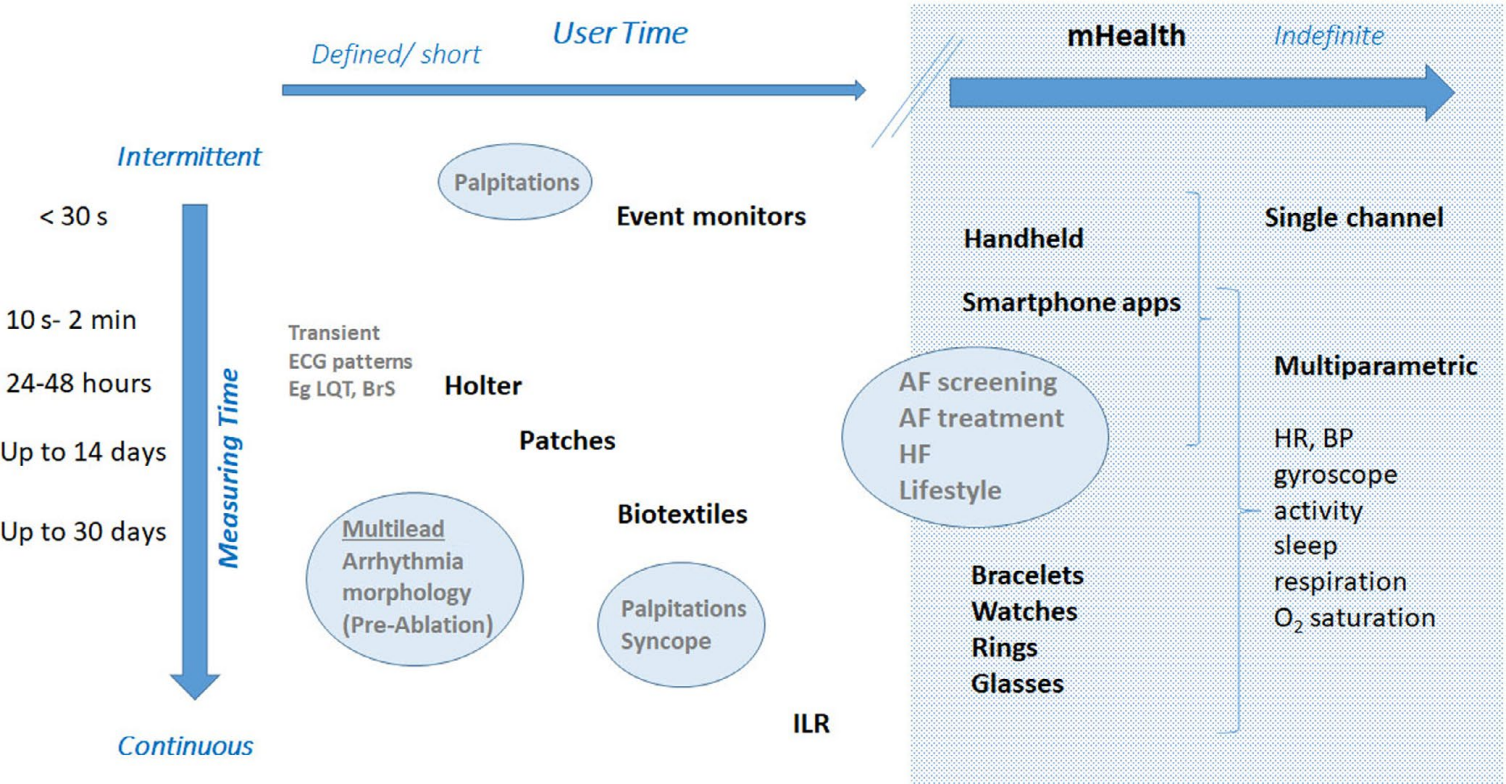
mHealth devices for arrhythmia monitoring according to indications. Traditional wearable monitors are used for defined, short periods of time. Advantages are continuous monitoring and ability to use multiple leads that may be important for arrhythmia differentiation. These have been used historically for evaluation of palpitations, syncope, and defining QRS morphology. mHealth extends monitoring time indefinitely, to be defined by the user, and to the possibility of monitoring other parameters simultaneously with the ECG and linking to machine learning. Typically, mHealth utilizes single‐channel ECG or derived heart rate, and discontinuous monitoring. AF—atrial fibrillation, BP—blood pressure, BrS—Brugada syndrome, HF—heart failure, HR—heart rate, ILR—implantable loop recorder, LQT—long QT.

This is the cornerstone diagnostic method, and the choice of technique and time frame depend on whether symptoms (e.g., palpitations, syncope) are present and how often they occur (Figure 2). Since the XXI century has become the era of the AF epidemic, the emphasis has shifted to screen for asymptomatic patients at high risk of developing AF or in those with cryptogenic stroke, to enable early treatment with the hope of preventing stroke and other serious complications. Novel tools expand the time window in which information can be gathered and overcome existing limitations with traditional methods, that is, intermittent physical examination or ECG for the detection of a largely asymptomatic arrhythmia.

- Conventional ambulatory ECG devices with “continuous” or “intermittent” recording abilities (e.g., Holter, mobile cardiac telemetry (MCT)) increase the diagnostic yield for suspected arrhythmias, but limitations such as inadequate duration of monitoring, insufficient sensitivity or specificity for AF detection, cost, and patient discomfort and inconvenience remain important implementation barriers. Further details on these conventional systems are available in a prior expert consensus statement (*Steinberg et al., 2017*).
- Implantable loop recorders (ILRs) continuously monitor cardiac rhythm, similar to traditional external loop recorders, but only record an ECG shortly before and after activation by either the patient or by an automated algorithm. The total monitoring period is limited only by battery longevity (ca. 2‐5 years). Newer devices have dedicated algorithms resulting in increased interest in their use for AF detection, especially after cryptogenic stroke. Several approved ILR devices are available (*Musat, Milstein, & Mittal, 2018, Sakhi, Theuns, Szili-Torok, & Yap, 2019, Tomson & Passman, 2015*), and several studies have been performed to evaluate the diagnostic accuracy of these devices (*Ciconte et al., 2017, Hindricks et al., 2010, Mittal et al., 2016, Nolker et al., 2016, Sanders et al., 2016*). Since ILRs are invasive and costly, some functions may shift to mHealth.

### 2.2 New mHealth‐based modalities for arrhythmia monitoring

These can be divided into technologies that:

- Record ECG tracings (single or multilead, in intermittent or continuous format, of various durations).
- Use non‐ECG techniques such as pulse photoplethysmography (PPG).

mHealth tools permit indefinite monitoring and widen application to a range of conditions and patient populations. There has been rapid development and integration of diagnostic sensors into consumer devices such as smartwatches, fitness bands, and smartphones. However, validation of their notified data (or underlying algorithms) and mechanisms for professional review (as established for CIEDs and MCTs) are scant, if at all (See Section 7). This is open to risks of not detecting significant events and/or overtreating—for example, false‐positive episodes of AF—if not confirmed by expert physicians.

#### 2.2.1 ECG‐based

Among these, handheld and patch systems have undergone the most extensive validation.

##### 2.2.1.1 Handheld devices

Several stand‐alone handheld devices operate without additional hardware. These devices with two or three ECG electrodes on either side generate short, 30 sec to 1 minute, single or multilead ECG recordings. Some of them display ECG tracings on a monitor. Most of these devices are equipped with dedicated automatic algorithms for detection of arrhythmias and usually focus on AF. Recognition of AF is usually based of the analysis of RR interval irregularity. The devices can store ECG tracings, which can be uploaded to a computer for review and are usually available for physicians via web‐based platforms. Studies across diverse populations have documented the diagnostic accuracy of handheld devices in detection of AF by short‐term rhythm monitoring (*Desteghe et al., 2017, Doliwa, Frykman, & Rosenqvist, 2009, Hendrikx, Rosenqvist, Wester, Sandstrom, & Hornsten, 2014, Kaasenbrood et al., 2016, Poulsen et al., 2017, Svennberg et al., 2017, Tavernier et al., 2018, Tieleman et al., 2014, Vaes et al., 2014*) (Table 2).

**Table 2:** Exemplary validation studies for various mHealth technologies

	Device	Author	n	Setting	Comparator	Sensitivity (%)	Specificity (%)	Requires ECG confirmation
	Pulse palpation	Cooke, Doust, and Sanders (2006)	2385	Meta‐analysis	12‐lead ECG	94	72	+
Handheld devices	Zenicor	Doliwa, Frykman, and Rosenqvist (2009)	100	Outpatient cardiology clinic	12‐lead ECG interpreted by cardiologist	96	92	
	MyDiagnostick	Tieleman (2014)	192	Outpatient cardiology clinic	12‐lead ECG interpreted by cardiologist	100	96	
	Omron HCG‐801	Kearley et al. (2014)	999	Primary care practices	12‐lead ECG interpreted by cardiologist	94.4	94.6	
	Merlin ECG event recorders	Kearley et al. (2014)	999	Primary care practices	12‐lead ECG interpreted by cardiologist	93.9	90.1	
Smartphone ECG device	AliveCor Kardia Mobile	Lau et al. (2013)	204	Recruited patients	12‐lead ECG interpreted by cardiologist	98	97	
Smartphone device PPG	CardioRhythm iPhone	Chan et al. (2016)	1013	Primary care clinic	Single‐lead AliveCor ECG	93	98	+
	PULSE‐SMART App	McManus et al. (2016)	219	Patients undergoing cardioversion	12‐lead ECG or 3‐channel telemetry	97	94	+
	FibriCheck App	Proesmans et al. (2019)	223	Primary care practices	12‐lead ECG	95	97	+
Smartwatch ECG	KardiaBand automated algorithm	Bumgarner et al. (2018)	112	Patients undergoing cardioversion	12‐lead ECG	93	84	
Blood pressure device	Microlife	Wiesel, Fitzig, Herschman, and Messineo (2009)	405	Cardiology outpatients	12‐lead ECG	95, 97 for one or 3 measurements, respectively	86, 89 for one or 3 measurements, respectively	+

##### 2.2.1.2 Wearable patches

Traditional cable/wire‐based devices increasingly have been displaced by solutions with electrodes embedded in adhesive patches. Commercially available patches can be worn up to 14 days (*Barrett et al., 2014, Turakhia et al., 2013*). Unlike adhesive electrodes for lead‐based systems, the water‐resistant patches are not removed during the monitoring period leading to greater wear time, more analyzable data, and no lead reversal errors. The cutaneous patch monitors are typically single‐use and continuously or intermittently record single‐lead electrocardiography. Most have an integrated button to mark the timing of symptoms on the recorded rhythm trace. After the monitoring period, the device is returned to the manufacturer for data extraction, analysis by a proprietary algorithm, and further secondary analysis of potential arrhythmias by medical technicians. A diagnostic report is sent to the treating physician. This process may be associated with delays of several weeks.

Although such patches only record a single‐lead ECG, a high agreement (P<.001) has been demonstrated compared to multi‐lead Holter monitors for identifying AF events and estimating AF burden (*Barrett et al., 2014, Rosenberg, Samuel, Thosani, & Zimetbaum, 2013*). As the patch has no external leads, it is perceived to be more comfortable to wear compared to conventional Holter monitors, with 94% of the patients preferring the patch over the Holter (*Barrett et al., 2014*). In addition to the validation studies, the feasibility of two‐week continuous monitoring to identify AF in an at‐risk patient population has been examined by *Turakhia et al. (2015).* It has also been used successfully to determine the prevalence of subclinical AF in the general population (*Rooney et al., 2019*).

Newer patch‐based systems add near‐real‐time analytics and by transmitting data continuously to the cloud. This may facilitate more rapid data collection and diagnosis. Multiparametric monitoring may be enabled with a patch worn for up to 3 months *(Stehlik et al., 2020)*.

##### 2.2.1.3 Biotextiles

Textile‐based systems for ECG monitoring were initially designed to ensure patients’ comfort during daily activities and address the needs of active patients. These vests and elastic bands adapt easily to patients’ movements that is particularly important for those performing physical activities that might be limited by the presence of wires. These biomedical devices capture the electrocardiographic signal via electrodes integrated into the garment that enables noninvasive acquisition of ECG signal up to 30 days. Single/multilead selection (up to full 12‐leads) and event activation are available. ECG signals can be stored in memory cards and analyzed afterward as well as transmitted in real time via Bluetooth to a smartphone (and from there to a cloud‐based platform), along with other signals including accelerometer and global positioning system (GPS). Other than ECG, some devices provide data on activity intensity, respiratory function, and sleep quality. Automatic analysis with manual verification is possible. Several systems for ECG monitoring based on electrodes incorporated into garments have been introduced into market. Some of them acquire signal from chest belts. Maintaining power presents a challenge. These systems have been tested in athletes, in patients with cryptogenic stroke, and in those with pacemaker‐detected AHRE (*Eliot, Hamlin, & Lizamore, 2019, Eysenck, Freemantle, & Sulke, 2019, Fabregat‐Andres et al., 2014, Feito, Moriarty, Mangine, & Monahan, 2019, Pagola et al., 2018*).

The wearable cardioverter‐defibrillator transmits 2‐channel ECG data to an online patient management database allowing for remote monitoring of high‐risk patients. Recent incorporation of heart sound evaluation that may predict HF decompensation will be tested in a prospective trial (*HEARIT‐Reg trial ClinicalTrials.gov Identifier: NCT03203629).*

##### 2.2.1.4 Smartphone and smartwatch‐based devices

**Figure 3:**
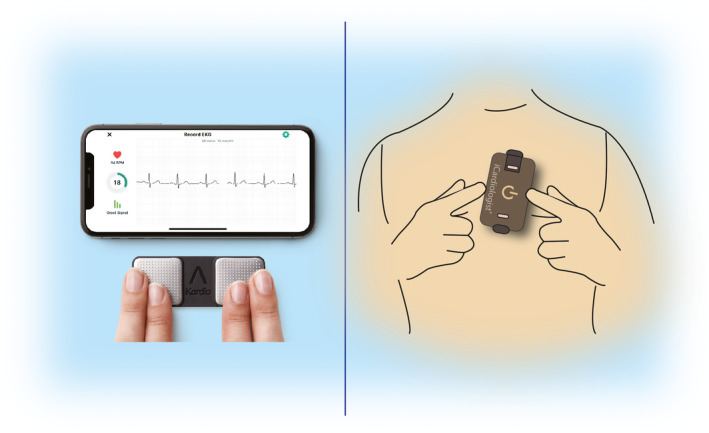
ECG mobile applications. Left—fingertip recordings; Right—card pressed to the chest.

More recently, nonwearable solutions coupled with the smartphone have emerged. These devices (Table 2 and *Varma et al., 2020*) allow the user to perform a “spot check” single‐lead ECG strip, usually of up to 30 seconds or longer by placing a finger of each hand on the two electrodes, usually located on the phone case or external card (Figure 3). The ECG electrical signal is transmitted wirelessly to a smartphone with an integrated interpretation app. The tracings can be reviewed on the smartphone, electronically stored, or transmitted for review by the user’s provider if desired. These have been directed largely to AF.

**Figure 4:**
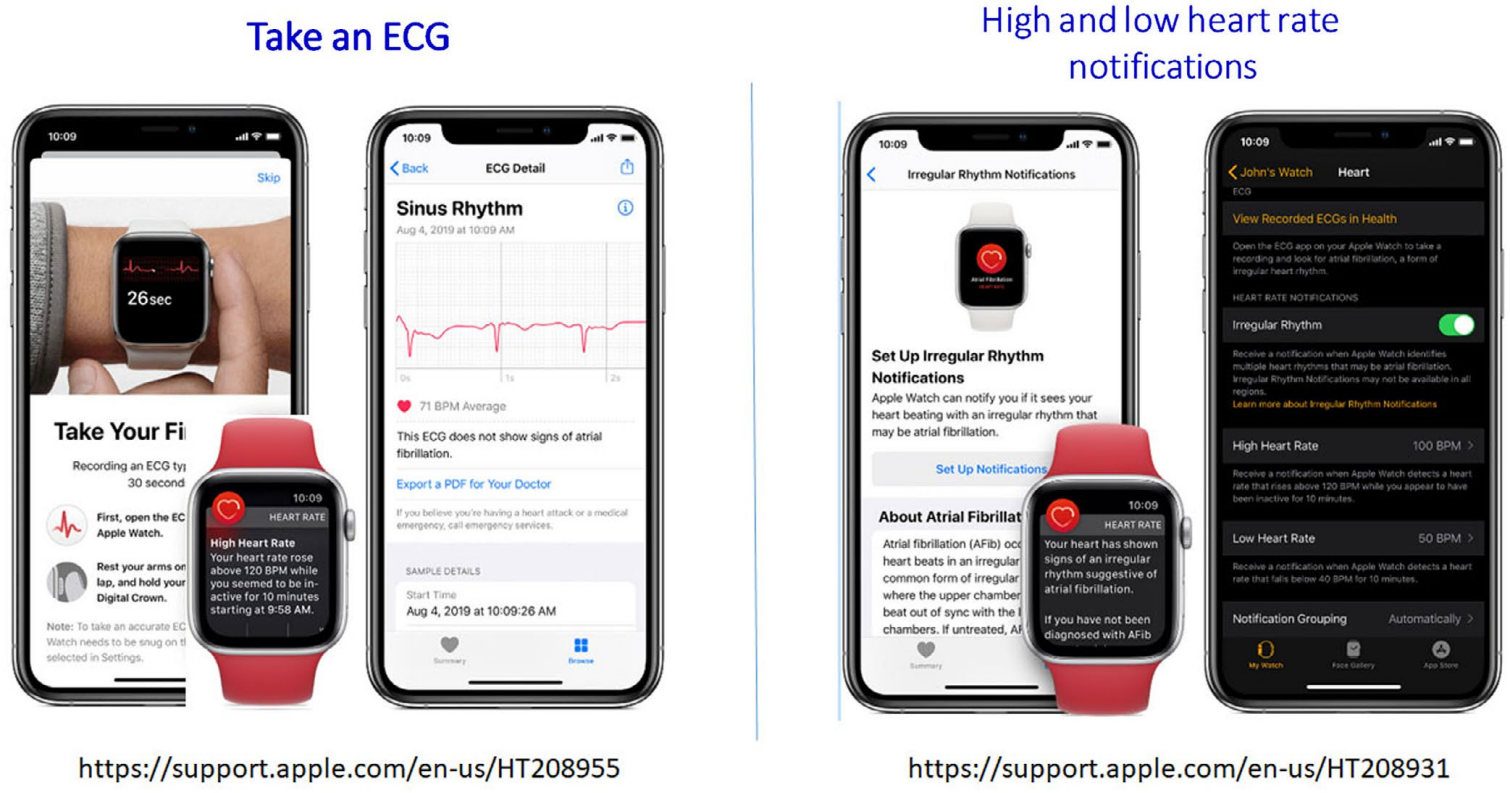
Apple Watch.

Automated algorithms can label the recording as “Possible AF” on the basis of criteria for the presence and absence of a P wave and the irregularity of the RR interval; “Normal” or “Sinus Rhythm” and “Unreadable” when the detector indicates there was too much interference for an adequate recording, whether from too much movement, or poor contact between the electrodes and the patient’s skin. Several versions of the AliveCor’s automated algorithms have been evaluated (*Chan et al., 2016, Chan et al., 2017, Desteghe et al., 2017, Lowres et al., 2014, Tarakji et al., 2015*), and the device has been tested as a screening tool in at‐risk populations (*Halcox et al., 2017, Lowres et al., 2014*). In Apple watch, the algorithm is effective when the heart rate is between 50 and 150 bpm, there are no or very few abnormal beats, and the shape, timing, and duration of each beat is considered normal for the patient (Figure 4).

Sensitivity and specificity depend on the software (which can be calibrated to higher sensitivity or higher specificity), the population studied (e.g., elderly have more tremor and/or difficulty in holding the device leading to more unreadable tracings), and the prevalence of AF in the population. It indicates that use of such device always requires proper evaluation for every intended use case. There is also an accessory band for a smartwatch to allow ECG recording. The single‐lead ECG with automatic AF detection is recorded by touching the band’s integrated sensors that transmit data to a watch application. Recently, a new 6‐lead case has been developed, allowing for 30 second recording of all 6 limb leads by touching each of the three electrodes. Also the QT interval may be derived from this (https://cardiacrhythmnews.com/kardiamobile‐6l‐can‐be‐used‐to‐measure‐qt‐duration‐in‐covid‐19‐patients/ (*Chung & Guise, 2015, Garabelli et al., 2016*). Information is limited; however, on how parameters such as QTc measured on a single‐ (or limited number) lead ECGs can reliably substitute for 12‐lead ECG information. In one study, QT was underestimated by smartphone single‐lead ECG (*Koltowski et al., 2019*). Preliminary data indicate ability for ST monitoring for ischemia (Figure 3, Section 4.1).

Such devices may be used by clinicians as a point‐of‐care device to obtain an interpretable rhythm strip in place of a 12‐lead ECG. In addition, patients may use these devices for ad hoc or routine evaluation of their rhythm in a home environment. The ECG data can be instantaneously transmitted for automated interpretation with the ability of the consumer to request a physician overread for a surcharge.

- Limitations
  - Single‐lead devices, particularly when used by an active person who may not be recumbent, relaxed, or still, may lead to substantial electrical or motion artifact. Noise‐free tracing may be more difficult for older patients or those with physical limitations (tremor, stroke, etc).
  - Although the interpretation algorithms typically have received regulatory oversight, these algorithms can frequently misclassify rhythms, calling sinus rhythm AF and vice versa, which could lead to potential harm without confirmation by a clinician. For example, in a recent study of a consumer ECG device to detect AF, a third of ECGs were unclassifiable by the device but could be classified by experts (Bumgarner et al., 2018) Therefore, some devices have limitations placed on them for diagnostic assessment. For example, the Apple Watch is unable to assess the ECG for AF if the heart rate is above 150 or below 50 bpm (https://www.apple.com/healthcare/docs/site/Apple_Watch_Arrhythmia_Detection.pdf) and is cleared by the U. S. Food and Drug Administration (FDA) only for use in persons without a diagnosis of AF (Figure 4) (https://support.apple.com/en‐us/HT208931, accessed January 2, 2020) (See Section 6) .
  - For consumer watches, ECG diagnosis is considered a prediagnostic pending medical verification and not designed to be acted on without clinician review.
  - ECG classification of other arrhythmias (premature ventricular complexes (PVCs), premature atrial complexes (PACs), ventricular tachycardia (VT)) is currently unavailable.

#### 2.2.2 Non‐ECG‐based

##### 2.2.2.1 Photoplethysmography

Consumer devices such as smartphones and smartwatches require accessories and often extra cost for conversion into rhythm monitoring tools. In contrast, the PPG technologies allow for the detection of arrhythmias using hardware already present on most consumer devices (smartwatches and fitness bands) through a downloadable application. PPG is an optical technique that can be used to detect AF by measuring and analyzing a peripheral pulse waveform. Using a light source and a photodetector, the pulse waveform can be measured by detecting changes in the light intensity, which reflects the tissue blood volume of a skin surface such as the fingertip, earlobe, or face (*Conroy, Guzman, Hall, Tsouri, & Couderc, 2017, McManus et al., 2013*). An automated algorithm can subsequently analyze the generated pulse waveform to detect AF. PPG avoids the instability and motion artifacts of ECG sensors and can be passively and opportunistically measured.

This technology has been applied for use with smartphones using the phone’s camera to measure a fingertip pulse waveform. Rapid irregularly conducted AF may produce variable pulse pressures that challenge detection (*Choi & Shin, 2017*). The performance of algorithms interpreting these PPG signals has been proven to be in high agreement with ECG rhythm strips (*McManus et al., 2013, McManus et al., 2016, Proesmans et al., 2019*). The smartphone‐based PPG applications have been utilized in at‐risk population to detect AF and as a screening tool in the general population (*Verbrugge et al., 2019*) (See Section 6).

The PPG technology has also been incorporated in smartwatches to measure heart rate and rhythm (*Dorr et al., 2019, Guo 2019*). Some have developed prototypes of a band that includes a single‐channel ECG, multi‐wavelength PPG, and tri‐axial accelerometry recording simultaneously at 128 Hz (*Nemati et al., 2016*), and others use a deep‐neural network based on PPG sensors to detect AF (https://www.mobihealthnews.com/content/study‐apple‐watch‐paired‐deep‐neural‐network‐detects‐atrial‐fibrillation‐97‐percent‐accuracy; https://mrhythmstudy.org). If PPG or optical sensors and detection algorithms can match the performance of ECG‐based rhythm assessment, delivery of AF care may be expected to change substantially and drive a radical departure from relying on an office or ambulatory ECG for ascertainment of AF.

##### 2.2.2.2 Oscillometry

Blood pressure (BP) measurements can be erratic when the pulse is irregular. This characteristic is utilized by automatic oscillometric BP monitors that derive heart rhythm regularity algorithmically (*Chen, Lei, & Wang, 2017*). Automated BP monitors have been used for opportunistic AF detection. Studies have shown that six devices from two manufacturers were reliable with sensitivities and specificities greater than 85% (*Kane, Blake, McArdle, Langley, & Sims, 2016*). These studies suggested that BP devices with embedded algorithms for detecting arrhythmias show promise as screening tools for AF, comparing favorably with manual pulse palpation. Such capability could be added to continuous BP recording devices (*Kario 2016*). One device identifies possible AF when at least two of three consecutive measurements show pulse irregularity. Several studies addressed the diagnostic accuracy (*Chan et al., 2017, Chen, Lei, & Wang, 2017, Gandolfo, Balestrino, Bruno, Finocchi, & Reale, 2015, Kearley et al., 2014, Marazzi et al., 2012, Stergiou, Karpettas, Protogerou, Nasothimiou, & Kyriakidis, 2009, Wiesel, Fitzig, Herschman, & Messineo, 2009, Wiesel, Arbesfeld, & Schechter, 2014)* and the feasibility of this device as a screening tool (*Chan et al., 2017, Omboni & Verberk, 2016, Wiesel & Salomone, 2017*).

The following have undergone preliminary study:

##### 2.2.2.3 Mechanocardiography

Mechanocardiography uses accelerometers and gyroscopes to sense the mechanical activity of the heart. The accuracy of this technology to detect AF using a smartphone’s built‐in accelerometer and gyroscope sensors was assessed in a proof of concept study (*Jaakkola et al., 2018*). A smartwatch (Sony Experia) was placed on the chest in supine patients to detect micro movements of the chest. Possibly, carrying this device in a pocket may have utility but is likely to be confounded by movement (e.g., walking) artifacts.

##### 2.2.2.4 Contactless video plethysmography

Noncontact video monitoring of respiration and heart rate have been developed less than 15 years ago (*Takano & Ohta, 2007, Verkruysse, Svaasand, & Nelson, 2008)*. In 2014, a pioneering article described the concept of contactless video‐based detection of AF (*Couderc et al., 2015*). Deep learning of a video of a person’s face can identify AF by examining irregularity of pulsatile facial perfusion (*Yan et al., 2018*). It is a monitoring technique extracting the photoplethysmographic‐like signals from a standard digital RGB video recording of the human skin and specifically of an individual’s face. The videoplethysmographic signal describes the absorption peak of ambient light by the hemoglobin from the facial skin. Several studies have been performed to develop a method that is sensitive enough to detect each cardiac pulse and provide insights into variability on pulse on a beat‐to‐beat basis. The HealthKam works using HUE color space from video cameras (*Dautov, Savur, & Tsouri, 2018, Tsouri & Li, 2015*) and can easily be integrated to any portable computer device with a camera (smartphone, tablet, etc.). By using mobile devices with cameras, the deployment of the technology is easy and scalable since it does not require the use and distribution of any physical devices. Such a system may change the approach to AF screening, which currently is only 1 patient at a time. High‐throughput AF detection from multiple patients concurrently using a single digital camera and a pretrained deep convolutional neural network (DCNN) was feasible in a pilot study (*Yan et al., 2020*).

##### Limitations

One requirement for these technologies is steady focus: Thus moving subjects present a challenge. It is important to avoid recording, sending, or communicating any video of the patient thus protecting privacy and dignity. Video‐based technologies in telemedicine have raised a new set of societal and ethical concerns that are being continuously re‐evaluated such as during the COVID‐19 pandemic. Issues regarding privacy, confidentiality, and legal and ethical obligation to treat are crucial factors to be considered when these technologies are deployed at larger scale (*Turakhia 2020*).

##### 2.2.2.5 Smart speakers

There are preliminary reports on using commodity smart devices to identify agonal breathing *(Chan, Rea, Gollakota, & Sunshine, 2019, Wang, Sunshine, & Gollakota, 2019).* Identification of abnormal heart rate patterns may be made possible by converting smart speakers into a sonar device with emission of in‐audible frequencies sound waves and receiving them to detect motion. These are not in consumer domain but potentially have wide scalability.

## 3 mHEALTH APPLICATIONS FOR ARRHYTHMIAS

Typically, most patients with palpitations and dizziness are evaluated using the various technologies reviewed in Section 2.1 (*Steinberg et al., 2017)*. Devices capable of recording at least one ECG lead allow the interpreting clinician to distinguish between wide‐ and narrow‐complex rhythms, bradycardia, and tachycardia, and thus distinguish between the various causative rhythms. Smart devices may be useful in pediatric patients (*Gropler, Dalal, Van Hare, & Silva, 2018)*.

### 3.1 Atrial fibrillation

**Figure 5:**
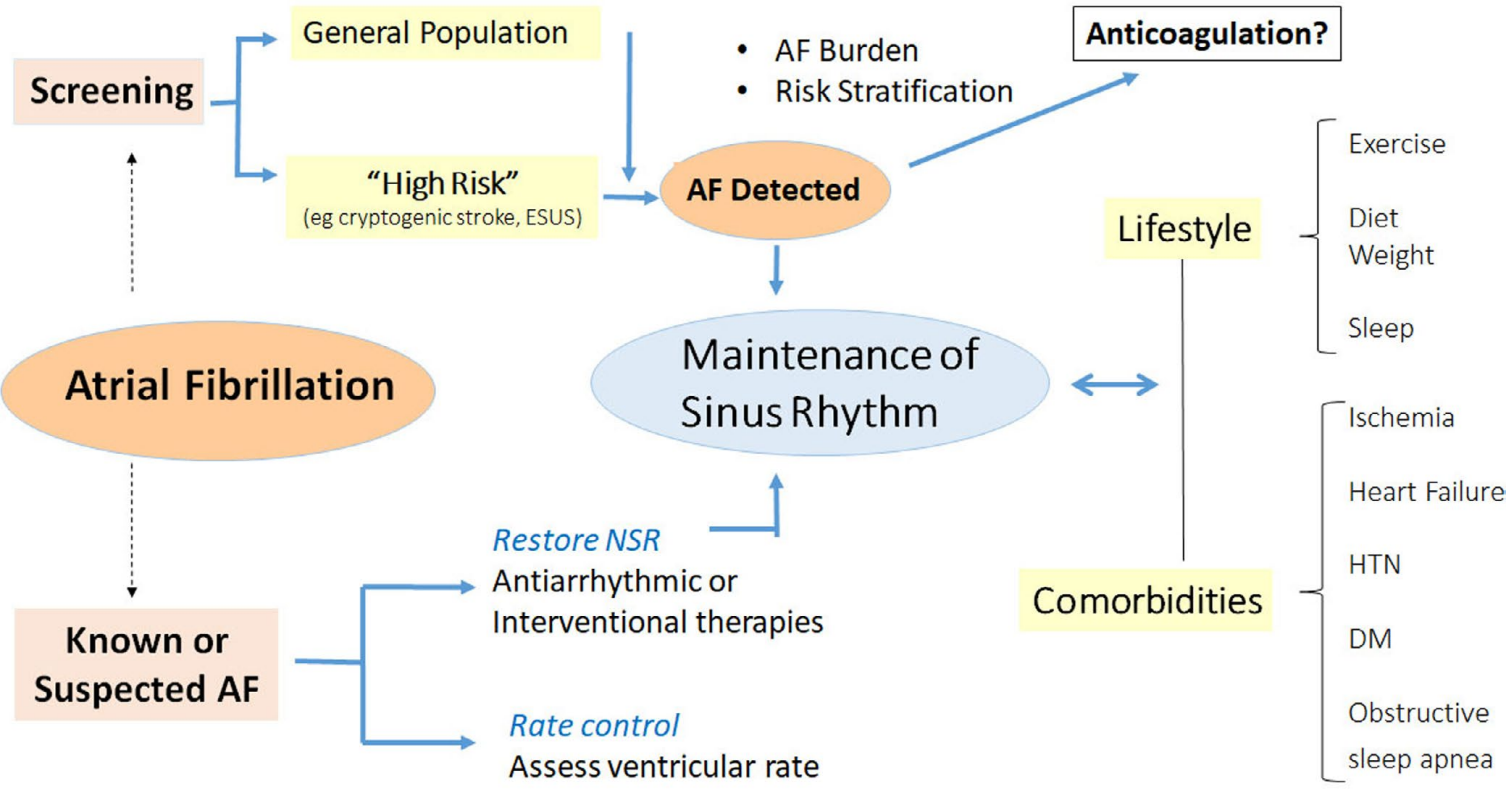
mHealth and AF. Applications include screening for AF in general or high‐risk populations, managing comorbidities and lifestyles important for prevention and control, as well as managing treatment of known AF. AF—atrial fibrillation, DM—diabetes, HTN—hypertension, NSR—normal sinus rhythm.

The disease is often intermittent and asymptomatic, which may delay diagnosis *(McCabe, Chamberlain, Rhudy, & DeVon, 2015, Strickberger, Ip, Saksena, Curry, Bahnson, & Ziegler, 2005,* *Verma et al., 2013*), lead to incorrect estimation of AF burden (*Boriani et al., 2015, Garimella et al., 2015*), and pose management challenges to healthcare services, thereby exposing the patient to the consequences of untreated AF. New digital health and sensor technologies have the potential for early identification of AF, opening up opportunities for screening, which then can be tied to evidence‐based management. These may be directed to several broad groups: for screening the general population or managing the already diagnosed, for following responses to treatment, and increasingly to managing comorbidities and lifestyle modification (See Section 4) (Figure 5). mHealth mechanisms may facilitate understanding the relation between AF burden, its progression, and cardiovascular risk (*Wong et al., 2018*).

#### 3.1.1 Undiagnosed atrial fibrillation identification

Classical epidemiological data point to the notion that early identification of AF has the potential to improve morbidity and possibly mortality. (1) AF is associated with a 5‐fold increased risk of stroke (*Wolf, Abbott, & Kannel, 1991*) and doubled mortality (*Kirchhof et al., 2016*); (2) The prevalence of undiagnosed AF is at least 1.5% for patients > 65 years (*Orchard et al., 2018*); (3) In about a quarter of all AF‐related strokes, the stroke is the first manifestation of the arrhythmia (*Friberg et al., 2014*) while other AF patients present first with congestive HF; (4) Stroke risk is independent of symptoms (*Xiong, Prioietti, Senoo, & Lip, 2015*); (5) Diagnosis often requires repeated or prolonged ECG monitoring; and (6) Oral anticoagulants (OACs) are highly effective in reducing the risk of cardioembolic stroke, mortality, and possibly dementia in the setting of AF (*Ding & Qiu, 2018, Friberg & Rosenqvist, 2018*).

Atrial fibrillation identification depends on factors having to do with the arrhythmia itself, that is the combination of AF prevalence and density (*Charitos et al., 2012*), and factors associated with detection such as the frequency and duration of monitoring and diagnostic test performance (*Ramkumar et al., 2018).* Several studies including patients with variable stroke risk factors have used mHealth technologies to identify undiagnosed AF (Tables 2 and 3), but these may require gold‐standard ECG confirmation.

**Table 3:** Selected screening studies for atrial fibrillation using newer technologies

	Device	Author, year	Setting	Inclusion criteria	N	Mean age (yrs)	Duration of monitoring	New AF detection (%)
Handheld ECG device	Zenicor SL	Berge et al. (2018)	Norway systematic	Age 63‐65 yrs,_ CHADS‐VaSC≥2 (M) or ≥3 (F)	1510	64	10 sec Twice daily for 2 weeks	0.9%
	Zenicor SL	Svennberg et al. (2015)	Sweden systematic	Age 75 to 76 yrs,	7173	75	10 sec Twice daily for 2 weeks	3.0%
	Zenicor SL	Engdahl, Andersson, Mirskaya, and Rosenqvist (2013)	Sweden systematic	Age 75 to 76 yrs, + CHADS2 risk score≥2	403	75	10 sec Twice daily for 2 weeks	7.4%
	Zenicor SL	Kemp Gudmundsdottir et al. (2020)	Sweden systematic	Age 75‐76 yrs + NTproBNP≥125 ng/l	3766	75	10 sec Twice daily for 2 weeks	4.4%
	Zenicor SL	Doliwa et al. (2012)	Sweden Postdischarge	Recent ischemic stroke/TIA and no prior AF	249	72	10 sec 30 days	4.8%
	My Diagnostick	Tieleman et al. (2014)	Netherlands	Influenza vaccination	676	74	1 min	1.6%
	My Diagnostick	Kaasenbrood et al. (2020)	Netherlands Primary care Opportunistic	Age>65 years	919		1 min	1.43%
	My Diagnostick SL	Tavernier et al. (2018)	Belgium Geriatric ward	Geriatric	252	84	Daily 1 min during hospitalization (median 5 )	13%
ECG Patch	ZioPatch iRhythm	Turakhia et al. (2015) (STUDY‐AF)	US	M, age≥65 yrs and ≥risk factors	75	69	Two weeks continuous	5.3%
	ZioPatch iRhythm	Steinhubl et al. (2018)	US National health plan members	Age≥75 yrs or M>55/F>65 years+risk factors	2659	72	Continuous 4 weeks	2.4%
	Zio XT Patch	Rooney et al. (2019) ARIC study	US Community surveillance study	No prior AF	386	79	Continuous 2‐4 weeks	2.5% (2 weeks) 4% (4 weeks)
	Zio Patch	Heckbert et al. (2018) Multi‐Ethnic Study of Atherosclerosis	US Community surveillance study	No prior AF	804	75	Continuous 2‐4 weeks	4% (AF/AFL)
Smartphone ECG‐based	AliveCor Kardia Mobile SL	Lowres et al. (2014) (SEARCH‐AF)	Australia Pharmacy Opportunistic	Age≥65 years	1000	76	30 sec	1.5%
	AliveCor Kardia Mobile SL	Chan et al. (2016)	Hong Kong Outpatient clinic	Age ≥65 years or HTN/diabetes	1013	68	30 sec	0.5%
	AliveCor KardiaMobile SL	Halcox et al. (2017) (REHEARSE AF)	UK Randomized trial	Age≥65 years +CHA_2_DS_2_‐VASc≥2	1001	73	30 sec Twice weekly for 1 year	3.8%
Smartphone device PPG‐based	Cardio Mobile app	Chan et al. (2016)	Hong Kong Outpatient	Age years ≥65 or HTN	1013	68	30 sec	0.5%
	Huawei wristband (Honor Band 4) or Huawei Watch	Guo et al. (2019)	General population across China	Age > 18 yrs	187,912	35	≥14 days	0.23%
Smartwatch	Apple smartwatch, iPhone app	Perez et al. (2019)	General population across USA	Age > 22 yrs	419,297	41	Median 117 days	0.52% irregular heart rhythm

Abbreviations: AF, atrial fibrillation; AFL, atrial flutter; F, females; HTN, hypertension; M, males; TIA, transient ischaemic attack; Yrs, years.

##### Accuracy

The positive predictive value of an AF event will differ according to pretest probability of AF in a given population (e.g., those with an established diagnosis or one or more risk factors). This is especially relevant to “healthy consumers.” Many technologies to identify AF are readily available directly to those without defined disease and are not deployed as individual or public health interventions. Rather, consumers who possess these technologies, such as smartwatches or smartphone‐connected ECG recorders, opt into the use of these technologies. Therefore, consumer‐driven AF identification is not the same as healthcare‐initiated AF screening. AF identification by these devices requires confirmation, since these AF screening tools have variable specificity (Table 2), raising the potential of a high false‐positive rate in a low prevalence population, and risks of unnecessary treatment.

There have been almost 500 studies assessing accuracy of mHealth devices for AF detection, as described in recent systematic reviews (*Giebel & Gissel, 2019, Lowres et al., 2019, O’Sullivan et al., 2020*). Their capabilities varied according to technologies utilized, settings, and study populations. Two large‐scale screening trials were reported recently (See Section 6).

##### Outcomes

No large outcome trial of screen detected AF and hard endpoints of stroke and death has been conducted as yet.

Although an incidental diagnosis of AF seems to be associated with increased risk of stroke and protection by OAC therapy (*Freedman, Potpara, & Lip, 2016, Martinez, Katholing, & Freedman, 2014, Tsivgoulis et al., 2019*), clinical trials to determine any benefit for opportunistically detected AF have not yet been completed but are underway (*Gudmundsdottir et al., 2020, Steinhubl et al., 2018, Svennberg et al., 2015, Heartline study* https://www.heartline.com). This effort addresses the concern that AF detected by screening may identify inherently lower‐risk patients so that efficacy of anticoagulation (and its risk/benefit ratio) requires recalibration. This is necessary prior to issuance of any recommendations. (Currently, no consensus exists yet on how to treat these arrhythmias, even in those with high CHA_2_DS_2_‐VASc scores).

The European and American guidelines do recommend opportunistic screening for early identification of undiagnosed AF in patients aged ≥65 years (*Freedman et al., 2017, January et al., 2019, Kirchhof et al., 2016*). On the other hand, the U. S. Preventive Services Task Force has presently given an “insufficient” recommendation for systematic screening for AF with electrocardiograms (*Jonas et al., 2018*).

#### 3.1.2 Targeted identification in high‐risk individuals

##### Cryptogenic stroke/TIA

Up to one‐third of ischemic strokes is attributed to AF mediated embolism to the brain (*Hannon et al., 2010*). Further, the risk of recurrent thromboembolism is high if AF is left undetected and untreated (*Furie et al., 2012, Kolominsky‐Rabas et al., 2001*). Hence, prolonged monitoring for AF poststroke has been recommended in recent guidelines (*January et al., 2019, Kirchhof et al., 2016, Schnabel et al., 2019*). Detection of AF poststroke depends not only on the monitoring device used and the duration of the monitoring period, but also on stroke type and patient selection; thus, the results of AF detection have been heterogenous *(Kishore et al., 2014, Sanna et al., 2014, Zungsontiporn & Link, 2018*). A meta‐analysis showed that a stepwise approach to AF detection in poststroke patients led to AF detection in 23.7% of patients (*Sposato et al., 2015*), while a combined analysis of two randomized and two observational studies showed a 55% reduction in recurrent stroke following prolonged cardiac monitoring (*Tsivgoulis et al., 2019*). However, the optimal AF duration threshold for initiating anticoagulation is currently unknown and may be lower in a poststroke population compared to those with fewer cardiovascular risk factors (*Kaplan et al., 2019*).

The risk of undiagnosed AF and other sources of thrombi has been considered high in embolic strokes of unknown source (ESUS), prompting studies that evaluated whether empiric NOAC therapy is more effective than antiplatelet therapy without a requirement of AF detection. Two of these studies, NAVIGATE ESUS (*Hart et al., 2018*) and RESPECT‐ESUS (*Diener et al., 2018*), have not shown a reduction in recurrent stroke in patients receiving NOACs. It should be emphasized that the mere detection of AF after ESUS is not necessarily proof of positive causation. A third study is ongoing, including patients with suggested atrial myopathy (enlarged atria, increased levels of NT‐proBNP, or enlarged P waves) (*Kamel et al., 2019*).

These findings underscore the need for AF detection prior to initiation of OAC therapy in patients with cryptogenic stroke, ESUS, or ischemic stroke of known origin, and mHealth devices can ease the process of detection (*Zungsontiporn & Link, 2018*). The threshold of AF burden may very well differ in patients who have had a suspected cardioembolic event and those who have not (*Kaplan et al., 2019*).

##### Other high‐risk individuals

The key to making AF identification feasible, efficient and clinically valuable is the selection of patients with an increased likelihood of harboring undiagnosed AF, rather than general screening in unselected populations. mHealth ECG recorders can facilitate frequent brief (e.g., 30 seconds) recordings over prolonged periods of time by the very ubiquity of devices (including smartphone‐based apps or watches). These devices are particularly well suited to capture intermittent or nonpersistent arrhythmias; however, it is likely that frequent sampling would be necessary to capture infrequent paroxysmal AF and even daily “snapshot” ECG monitoring may miss half of AF episodes (*Charitos et al., 2012, Yano et al., 2016)*. AF burden, increasingly recognized as a powerful independent predictor of stroke (*Chen et al., 2018)*, though accurately measured by implanted devices (*Varma, Stambler, & Chun, 2005*), cannot be readily calculated from intermittent ECG data. The use of smartwatches with passive intermittent surveillance using PPG monitoring plus ECG confirmation may be a more effective screening tool and is currently being evaluated (*Heartline study* https://www.heartline.com).

Formal screening with mHealth ECG recordings has yielded meaningful incidences of newly diagnosed AF, statistically greater than if diagnosis relied only on the office ECG (Table 3). The yield generally is enhanced by the presence of risk factors, such as older age and higher CHA_2_DS_2_‐VASc scores. Several studies (*Chan & Choy, 2017, Chan, Wong, et al., 2017, Proietti et al., 2016*) screened untargeted populations, and all yielded new AF diagnoses at a rate under 1%. By focusing on older patients (75‐76 years of age) at greater risk, Swedish studies identified new AF in 3% of study participants, and up to 7.4% when additional risk factors beyond age were required (*Engdahl, Andersson, Mirskaya, & Rosenqvist, 2013, Gudmundsdottir et al., 2020, Svennberg et al., 2015*). *Lowres et al* in a patient level meta‐nalysis found that new AF detection rate increased progressively with age from 0.34% for <60 years to 2.73% ≥85 years. Importantly, the number of subjects needed to screen to discover AF meeting indications for anticoagulation was 1089 for subjects <60 years but 83 ≥65 years.

#### 3.1.3 Diagnostics in people with established atrial fibrillation

mHealth has important implications for the care of those already diagnosed with AF. Several key characteristics of AF can be measured with long‐term continuous or near‐continuous monitoring, and the information gained may provide valuable information for patient management.

Furthermore, while several studies succeeded in establishing the sensitivity and specificity of novel devices for the detection of AF, no study to date has yet evaluated the utility of an mHealth intervention in affecting clinical outcomes. The iPhone Helping Evaluate Atrial Fibrillation Rhythm through Technology (iHEART), a single‐center, prospective, randomized controlled trial, and the Heartline study seek to accomplish this goal (*Caceres et al., 2020, Hickey et al., 2016,* https://www.heartline.com).

#### 3.1.4 Atrial fibrillation therapy

##### Atrial fibrillation burden

Current guidelines for anticoagulation are based principally on the presence of risk factors and a diagnosis of clinical AF, regardless of AF duration, symptomatology, or burden (*January et al., 2019*). This applies even if the AF has been quiescent for long periods or eliminated altogether as the result of rhythm control interventions including antiarrhythmic drugs, ablation, or risk factor modification (*January et al., 2019*). However, there is increasing recognition that AF burden matters; for example, paroxysmal events have less thromboembolic risk than persistent AF (*Chen et al., 2018*). This understanding has been extended during continuous monitoring from CIEDs which depict AF with high granularity, and first advanced the metrics of “AF days” and burden in terms of cumulative load (hours/day) and concentration (density of AF days) (*Varma, Stambler, & Chun, 2005*). This measure is likely to be important for understanding mHealth discovered AF.

##### CIEDS

AF burden can be characterized as %/time monitored, longest duration, and density. Retrieved data provide an insight into natural history and associated sequelae (*Healey et al., 2012, Kaplan et al., 2019, Van Gelder et al., 2017, Varma, Stambler, & Chun, 2005*). This led to oral anticoagulation intervention trials to determine the ability to reduce stroke on the basis of AF duration (*Lopes et al., 2017, Martin et al., 2015*). These suggest that a threshold exists below which the risk of thromboembolic stroke is low and risk–benefit ratio may not justify chronic administration of oral anticoagulants. For instance, CIED data indicate that short subclinical AF events have lesser risk than more prolonged (and therefore more likely to be symptomatic) events (*Al‐Turki, Marafi, Russo, Proietti, & Essebag, 2019*). Device‐detected, “subclinical” atrial high‐rate episodes (AHRE) lasting 6 minutes to 24 hours are associated with increased stroke risk, but the absolute risk is considerably lower than expected based on risk factors alone (*Glotzer et al., 2003, Healey et al., 2012, van Gelder et al., 2017)*. Whether these require anticoagulation in high‐risk individuals is the subject of ongoing studies (*Kirchoff et al., 2017, Lopes et al., 2017, van Gelder et al., 2017*). Importantly, very short AF episodes (episodes in which both the onset and offset of AT/AF were present within a single EGM recording) were not associated with adverse outcomes (*Swiryn et al., 2016*) which may be important for mHealth monitoring.

##### mHealth

AF detection using digital health tools offers further insights in patients without indication for implantable devices. mHealth extends AF screening to younger patients without cardiovascular disease and thromboembolic potential may be low. Those with high AF burden (defined by ≥ 11.4%; mean duration 11.7 hours) detected on a 14‐day patch monitor had an increased thromboembolic event rate compared to those with lower AF burdens (*Go et al., 2018*). There remains significant treatment variation in use of OAC, especially for device‐detected AF (*Perino et al., 2019*). This may be due to a large clinical uncertainty regarding the optimal cutpoint, even though observational data indicate that OAC is associated with a decreased risk of stroke for episodes > 24 hours and possibly for episodes 6‐24 hours (*Perino et al., 2019).*

Currently, there are no prospectively validated cutpoints or risk models that incorporate AF burden into decision‐making for stroke prevention therapies.

Key knowledge gap:

- Identify characteristics (duration, episode number/ density) and risk factors that justify anticoagulation for mHealth detected AF.

##### Rhythm and Rate control

- *Rhythm* While we await data on OAC treatment for mHealth detected AF, the finding of the arrhythmia should initiate mHealth monitoring of NSR retention, QT intervals (important for those on some antiarrhythmic drugs (*Garebelli et al., 2016*), and discussion of cardiovascular risk factor modification and lifestyle changes, since AF coexists with comorbidities that may influence its occurrence and natural history (*See* *Section* *4*). Thus, alcohol reduction, treatment of OSA, moderate exercise, and weight loss have been shown to reduce AF burden (*Congrete et al., 2018, Kanagala et al., 2003, Pathak et al., 2015, Voskoboinik et al., 2020*).
- *Rate* While the primary goal of rate control is to minimize AF‐related symptoms, prolonged tachycardia can result in effort intolerance and/or tachycardia‐mediated cardiomyopathy while excessively low heart rate targets may increase the risk of bradyarrhythmias that result in symptoms and device implantation. The European Society of Cardiology recommends lenient resting heart rate targets (<100‐110), whereas the ACC/AHA/HRS guidelines recommend a target rate of <80 bpm. Often these targets are tailored to the individual patient based on symptoms and presence or propensity for HF. mHealth technologies can be used to assess ventricular rates during AF over long time periods and evaluate the effects of rate‐control therapies (*January et al., 2019, Kirchoff et al., 2016)*.

### 3.2 Sudden cardiac death

(See also section 4.1 Ischemic heart disease).

#### Ventricular arrhythmias

The use of mHealth technology to diagnose ventricular arrhythmias lags behind its application to AF (See section 3.1). Detection of symptomatic VT has been reported using the AliveCor cardiac monitor (AliveCor, San Francisco, USA) and SmartWatch (*Ringwald, Crich, & Beysard, 2019, Waks, Fein, & Das, 2015*). Sophisticated automated analysis of a 2‐minute PPG recording by the camera of a commercially available smartphone (iPhone 4S, Apple) can distinguish between AF, PACs, and PVCs from sinus rhythm, with a sensitivity of 0.733 and specificity of 0.976 for PVCs (*Chong, Esa, McManus, & Chon, 2015, McManus et al., 2016*). PVCs may challenge to PPG‐based systems, as many PVCs are nonperfusing (*Billet et al., 2019*). An ECG tracing is therefore essential in order to facilitate rhythm diagnosis and avoid misclassification of “slow PPG pulse rates” (bradysphygmia) simply as “bradycardia.”

#### Syncope

Syncope presents unique challenges for mHealth applications. While prolonged ambulatory monitoring using medical‐grade devices (wearable and implantable) has been the mainstay of cardiac rhythm diagnosis during episodes of syncope, user‐activated systems must either be activated by the patient during prodromal symptoms (if present and time permits) in anticipation of syncope, or else incorporate loop recording to allow postsyncope activation (*Steinberg et al., 2017*). This capability is not incorporated in currently popular consumer‐grade wearable devices. However, a randomized controlled trial of AliveCor versus usual care in participants presenting with palpitations or presyncope showed a faster and increased rate of detection of symptomatic arrhythmias in the intervention group, suggesting that at least in presyncope, patient‐activated rhythm detection using a commercially available mHealth device is productive (*Reed et al., 2019*). Rhythms reported by devices that rely on heart rates will likely require validation with a medical‐grade system to provide an ECG tracing during an event to allow determination of the causative rhythm.

There is a significant overlap between transient loss of consciousness and mechanical falls due to orthostatic intolerance, neurologic, or orthopedic problems. This is particularly disabling in elderly subjects and often unwitnessed (*Davis et al., 2010, Heinrich, Rapp, Rissmann, Becker, & Konig, 2010*). Mobile applications that combine analysis of heart rate monitoring together with fall detection, GPS positioning, video recording with display of patients’ surroundings, and the capability to send alerts either triggered by patients in case of symptoms or automatically in case of detected falls, may be useful.

#### Cardiac arrest

The detection and response to sudden cardiac arrest (SCA) is an area where mHealth applications may prove lifesaving. As rapid treatment for cardiac arrest has consistently been associated with improved survival, pre‐emptive identification of at‐risk persons, detection of cardiac arrests, alerting of nearby lay and professional first responders, and coaching or quality assurance in the performance of cardiopulmonary resuscitation (CPR) are ideally suited to the mHealth paradigm in societies where mobile smartphones are ubiquitous.

#### Prediction

It is possible that mHealth devices which continuously monitor heart rhythm and other physiologic data may be able to better predict impending SCA, even using measures which have not shown sufficient specificity or sensitivity when measured intermittently, such as heart rate variability (*Lee, Shin, Seo, Nam, & Joo, 2016*). However, such continuous monitoring is present already in CIEDs and has not yet proven to be sufficiently predictive to be clinically useful (*Au‐Yeung, Reinhall, Bardy, & Brunton, 2018*). Therefore, the prediction of SCA by mHealth devices, while a tantalizing prospect, remains to be realized.

#### Notification and reaction

Once cardiac arrest occurs, rapid identification is essential to trigger a response by emergency responders. Wearable devices that combine physiologic monitoring, GPS, and a method of communication with emergency services such as cellular service are well positioned to provide almost instantaneous alert as well as location information (*Kwon, Lee, Lee, Lee, & Park, 2018, Praveen Kumar, Amgoth, & Annavarapu, 2019*). An early device using a piezoelectric sensor to detect the pulse was capable of transmitting an alert to emergency medical system or other responders when a pulse was not detected and the watch (and thus the wearer) was still (*Rickard et al., 2011*). Preliminary reports indicate that smart speakers in commodity smart devices may be able to identify agonal breath patterns for sudden cardiac death detection *(Chan, Rea, Gollakota, & Sunshine, 2019).* Widespread diffusion of such technology to patients at elevated risk of SCA will be necessary before any potential benefits can be tested.

The ubiquity of mobile phones in society leads to more rapid notification of emergency services, and the possibility of a dispatcher gathering information from a bystander at the patient’s side and delivering instructions on care, such as CPR. This was associated with improved outcomes for a variety of emergencies (*Wu et al., 2012*). Notification of lay first responders in the vicinity of a cardiac arrest is also feasible with current technology. A blinded, randomized trial conducted in Stockholm, Sweden, demonstrated that such a system improved the rate of bystander CPR (*Ringh et al., 2015*). However, almost 10,000 volunteers were recruited over approximately 18 months, during which 667 activations occurred, emphasizing the large resources needed and the low rate of utilization of trained volunteers, even when alerted by mobile phone.

Whether a trained or novice bystander responds, mobile devices may be further useful to provide voice (or video) instructions from a dispatcher or from the device itself. Studies of prerecorded audio, live video, and animation‐based instruction have shown improvements in some aspects of CPR delivery and AED use, although technology continues to evolve (*Bolle, Scholl, & Gilbert, 2009, Choa et al., 2008, Merchant et al., 2010, You, Park, Chung & Park, 2008*). One limitation is that as such apps are unregulated, many do not convey current basic life support algorithms and may have poor usability (*Kalz et al., 2014*). In addition, delay in commencing CPR and in calling emergency services due to distraction of the rescuer by using an app is a concern (*Paal et al., 2012*).

Automated external defibrillator (AED) use in cardiac arrest is associated with improved survival, but AED use remains low (*Weisfeldt et al., 2010*). Mobile devices have the potential to increase this by assisting with the retrieval and use of AEDs. Multiple apps have been created to locate AEDs in the vicinity of the user, although with mixed results in simulations (*Sakai et al., 2011, Hatakeyama et al., 2018, Neves Briard et al., 2019*). Barriers include the accuracy of AED location databases, size of the user base, app interface, and the availability of multiple apps instead of a single validated regional, national, or international standard. An emerging approach to circumvent these limitations is the dispatch of an AED via a drone to the location of the cardiac arrest, which is expected to reduce time to defibrillation, especially in rural areas (*Boutilier et al., 2017*). Feasibility has been demonstrated (*Claesson et al., 2017)*.

#### Clinical trial

The complete chain from activation of citizen responders was tested in the Heartrunner trial (*Andelius et al., 2020*) in a region of almost 2 million inhabitants. Results showed that citizen responders arrived before emergency services 42% of out of hospital cardiac arrests, accompanied by a threefold increase in bystander defibrillation with a trend to improved 30‐day survival. Results were more pronounced when emergency arrival times were longer, for example, in rural areas.

## 4 COMORBIDITIES

**Figure 6:**
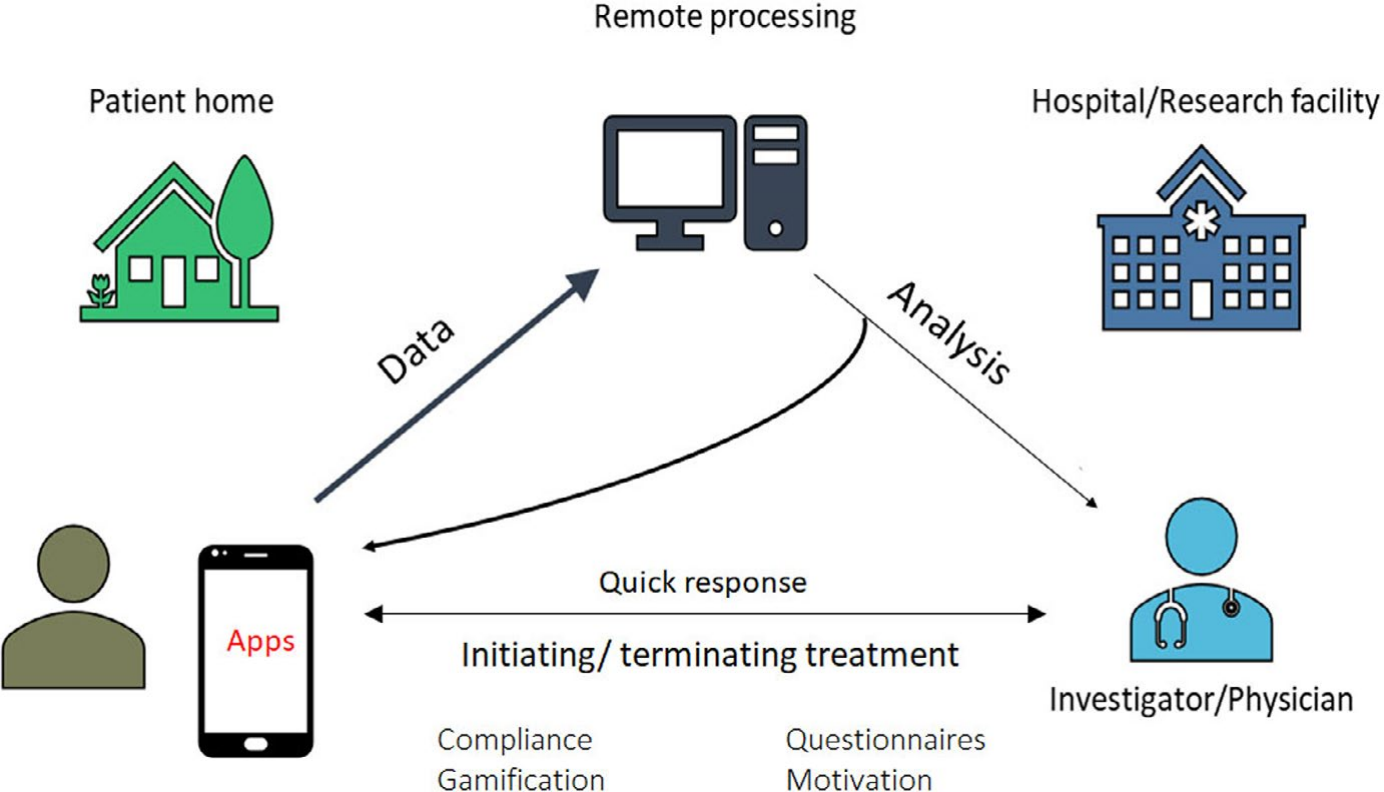
Digital applications can integrate patient relayed information of sensor and clinical information with automatic remote analysis, but also permit patients to receive advice and treatment adjustments from physicians directly.

A large proportion of arrhythmias are influenced by coexisting conditions. Their management may directly affect arrhythmia recurrence and outcome. Thus, lifestyle modifications and management of comorbid conditions (Figure 5) is becoming an objective of arrhythmia management (*Chung et al., 2020*) and received a Class 1 recommendation in most recent guidelines (*January et al., 2019)*. mHealth has significant potential for facilitating these interventions (Figure 6).

### 4.1 Ischemic heart disease

Early management (e.g., primary angioplasty) of acute ischemic syndromes may reduce infarct territory and ventricular arrhythmias, thereby improving outcome. AF after myocardial infarction worsens prognosis (*Pizzetti et al., 2001*).

#### At home

ST segment monitoring technology embedded in conventionally indicated ICDs when tested in a randomized cross‐over study suggested a reduction in the time from the onset of ischemia to presentation to hospital (*Gibson et al., 2019, Holmes et al., 2019*). The AngelMed Guardian system (Angel Medical Systems, Eatontown, New Jersey) is approved for use in the United States for patients with prior acute coronary syndrome (ACS) who remain at high risk for recurrent ACS. For lower‐risk patients, mHealth may improve symptom recognition and earlier presentation, that is, “symptom‐to‐door time” (*Moser et al., 2006*).

Wearable devices that continuously monitor physiologic data promise detection, and possibly pre‐emption, of the early stages of MI, by alerting patient and/or healthcare team early. A noninvasive device consisting of a three‐lead ECG linked wirelessly to a dedicated mobile device has recently been described (*Van Heuverswyn et al., 2019*). Three lead ECG tracings (as well as derived augmented limb leads) can be recorded with commercially available smartwatches (*Avila, 2019*). Limitations of this approach are the need for the patient or a bystander to possess the device or app, and be familiar with its use, before the onset of symptoms.

An emerging technology (www.heartbeam.com) uses a credit card sized device that is pressed against the user's chest (Figure 3). It collects ECG signals using a novel 3D vector approach. The signals are sent to the cloud, where they are analyzed and compared to the patient’s asymptomatic baseline reading. A proprietary algorithm combines the signal analysis with the patient’s history and reported symptoms. This information, along with a diagnostic recommendation and ECG waveforms, is sent to the patient’s physician, who makes a final determination and informs the patient This system is used by patients in the telehealth setting to assess whether chest pain is the result of an myocardial infarction.

#### Emergency teams

The next step of patient care involved transmission of ECGs by emergency responders in the field to hospitals for review and triage and was shown to result in shorter door‐to‐balloon time, lower peak troponin and creatine phosphokinase levels, higher postinfarction left ventricular ejection fraction, and shorter length of stay compared with control patients whose ECGs were not transmitted (*Clemmensen, Loumann‐Nielson, & Sejersten, 2010, Sanchez‐Ross et al., 2011*). This paradigm has now been widely implemented. Technical factors, such as transmission failure and lack of network coverage, are the main impediments to adoption of such systems.

#### Posthospital care

This is often confusing for patients, who often exhibit a poor understanding of their medications, follow‐up procedures, and future appointments (*Horwitz et al., 2013; Ziaeian, Araujo, Van Ness, & Horwitz, 2012*). This contributes to frequent hospital readmissions. Mobile technologies may enable individualized contact between patients and healthcare providers. Phone calls led to a modest improvement in medication adherence in patients with coronary artery disease in one large randomized controlled trial (*Vollmer et al., 2014*). Text messaging was shown to increase medication adherence and improved cardiovascular risk factors (*Chow et al., 2015; Unal, Giakoumidakis, Khan, & Patelarou, 2018*). Available evidence is limited by short‐term follow‐up and self‐reported adherence (*Shariful Islam et al., 2019*). Success may depend on personalized messages with tailored advice, the ability to respond to texts, timing messages to coincide with medication doses, higher frequency of messages, and the use of additional apps or websites (*Park, Beatty, Stafford, & Whooley, 2016*). Interoperability with the EMR may facilitate this approach.

#### Cardiac rehabilitation

This was shown to improve health outcomes among patients with heart disease, but is underutilized. The Million Hearts Cardiac Rehabilitation Collaborative aims to increase participation rates to ≥70% by 2022 (*Ritchey et al., 2020*). Mobile apps and linked sensors to measure heart rate, respiration rate, and exercise parameters may overcome traditional limitations of availability, cost, and convenience and be more acceptable to some patients (*Zwisler et al., 2016*). A randomized controlled trial center‐based and mobile rehabilitation found improved uptake, adherence, and completion with home‐based cardiac rehabilitation in postinfarction patients (*Varnfield et al., 2014*) (See also 4.2.2.)

### 4.2 Heart failure

Heart failure is widely prevalent, costly to manage, and degrades patient outcomes (*Benjamin et al., 2017, Albert & Estep, 2019*). HF may trigger AF and ventricular arrhythmias. Conversely, AF may precipitate HF. Remote monitoring of, for example, dietary and medication adherence (See Section 4.6.2), detection of arrhythmias (See Section 3), intercurrent ischemia (See Section 4.1), orthopnea, changes in heart rate, activity, and sleep (See Section 4.5) may enable remote adjustment of management to reduce emergency department visits and unplanned HF‐related hospitalizations. If scalable, remote monitoring coupled with mobile communication could prove to reduce costs associated with HF.

Despite promise, most large, multicenter randomized trials failed to demonstrate improved outcomes of remote monitoring in HF patients (Table 4) (*Boyne et al., 2012, Chaudhry et al., 2010, Dickinson et al., 2018, Koehler et al., 2011, Ong et al., 2016, Takahashi et al., 2012*). Combination algorithms based on multiple parameters may be valuable (*Ono & Varma, 2017*). One trial stands out. The TIM‐HF2 trial randomized HF patients to either remote patient management plus usual care or to usual care only and were followed up for over a year (*Koehler et al., 2018*). The results showed reduction in the combined endpoint of percentage of days lost due to unplanned hospitalization and all‐cause mortality. However cardiovascular mortality was similar between remote monitoring and standard care groups. Implanted devices that monitor pulmonary arterial pressure may be beneficial in select patients when used in structured programs (*Dickinson et al., 2018*). The positive findings of the CHAMPION trial (CardioMEMS Heart Sensor Allows Monitoring of Pressure to Improve Outcomes in NYHA Functional Class III Heart Failure Patients) trial and subsequent FDA approval has renewed interest in remote patient management for HF patients (*Abraham et al., 2016, Carbo et al., 2018, Desai et al., 2017*). This requires daily download of hemodynamic data and a prespecified medical treatment plan. An app is also available which illustrates patient compliance with monitoring, alerts the patient when transmissions are not received, shows medication reminders, and allows for medication reconciliation and titration.

**Table 4:** Randomized trials with neutral results based on external‐device remote patient monitoring (RPM)

Study name	Sample size	Study design and tested modality	Potential explanantion for lack of benefit
TIM‐HF (Koehler Circulation 2011)	N = 710 (355 on RPM)	Randomized trial of a Bluetooth‐enabled device designed to follow 3‐lead electrocardiography, BP, and weight	Participants had stable HF, so it may be that remote monitoring is not as effective in lower‐risk patients
Tele‐HF ( Chaudhry N Engl J Med 2010)	N= 1653 (826 on RPM)	Telephone‐based interactive voice response system with a higher risk population than in the TIM‐HF study	Patient adherence was poor, with <55% of the study subjects using the device 3 days per week by the end of the study. Interestingly, a smaller previous trial had shown benefit; this difference in results implies that how a technology is implemented might determine benefit
BEAT‐HF (Ong JAMA Intern Med 2016)	N = 1437 (715 on RPM)	Health‐coaching telephone calls with monitoring of weight, BP, HR, and symptoms in a high‐risk population with 50% rehospitalization rate	Nonadherence was the primary limitation, with only 61% of patients more than half‐adherent in the first 30 days
Mayo Clinic Study (Takahashi Arch Intern Med 2012)	N = 205 (102 on RPM)	Telemonitoring in a PC panel (various health conditions and not only HF) in the top 10% of Elder Risk Assessment Index managed with biometrics (BP, HR, weight, pulse oximetry, etc) plus daily symptom assessment. Video conference capability was present.	Abnormal telehealth data were directed to PC providers. It is unclear what action this drove. It might have caused the PC provider to direct the patient to an emergency department or a hospital. Could increased symptom surveillance actually increase healthcare utilization?
TEHAF (Boyne Eur J Heart Fail 2012)	N = 382 (197 on RPM)	Electronic device to assess symptoms and educate patients with HF. Abnormal symptoms directed to a monitoring nurse. Device tailored itself to patient’s knowledge.	Excellent adherence with use of the device. Planned and unplanned face‐to‐face HF nurse visits were higher in the control group. Event rates for both groups were lower than expected. Primary limitation appeared to be the excellent outcomes in the control group.
LINK‐HF (Stehlik, CIrc HF 2020)	N=100	Disposable multisensor chest patch for 3 months linked via smartphone to cloud analytics. Apply machine‐learning algorithm.	Pilot study, compliance eroded. However, this detected precursors of hospitalization for HF exacerbation with 76% to 88% sensitivity and 85% specificity.

Abbreviations: BP, blood pressure; HF, heart failure; HR, heart rate; PC, primary care; RPM, remote patient monitoring.

#### 4.2.1 Mobile technologies for managing heart failure

The concept of coupling remote monitoring and mobile cellular technologies is attractive for the HF community (*Carbo et al., 2018, Cipresso et al., 2012*). Heart rate (ECG), BP, and weight were the most frequently monitored parameters. Sensors that detect respiratory rate and pattern by detecting movement of the chest wall, via pressure, stretch, or accelerometry, may have applications in HF. Detecting breathing via microphone (sounds), change in impedance, or pulse oximetry are other possible means to monitor respiratory function. Some of these modalities could be integrated into smart clothing (*Molinaro et al., 2018*).

Some trials included also alert reminders of medication use, voice messages on educational tips, video education, and tracking of physical activity (See Section 4.6.1). Patients were mostly monitored daily and followed for an average of 6 months. A reduction was seen in HF‐related hospital days (*Carbo et al., 2018*). High rates of patient engagement, acceptance, usage and adherence have been reported in some trials but not others (*Chaudhry et al., 2010, Hamilton, Mills, Birch, & Thompson, 2018*).

Preliminary results using a disposable multisensor chest patch in the LINK‐HF study were encouraging (*Stehlik et al., 2020*), detecting precursors of hospitalization for HF exacerbation with 76% to 88% sensitivity and 85% specificity, 1 week before clinical manifestations.

#### 4.2.2 Hybrid telerehabilitation in patients with heart failure

Exercise training is recommended for all stable HF patients (*Piepoli et al., 2011, Ponikowski et al., 2016*). Hybrid cardiac telerehabilitation is a novel approach. Telerehabilitation is the supervision and performance of comprehensive cardiac rehabilitation at a distance, encompassing: telemonitoring (minimally intrusive, often involving sensors), teleassessment (active remote assessment), telesupport (supportive televisits by nurses, psychological support), teletherapy (actual interactive therapy), telecoaching (support and instruction for therapy), and teleconsulting and telesupervision of exercise training (*Piotrowicz, Piepoli, et al., 2016*). Various devices have been described, from heart rate monitoring (*Smart, Haluska, Jeffriess, & Marwick, 2005*) and transtelephonic electrocardiographic monitoring (*Kouidi, Farmakiotis, Kouidis, & Deligiannis, 2006*) to tele‐ECG‐monitoring via a remote device (*Piotrowicz, Zieliński, et al., 2015*) and real‐time ECG and voice transtelephonic monitoring (*Ades et al., 2000*).

Home‐based telerehabilitation was demonstrated to be safe, effective with high adherence among HF patients. It improves physical capacity (*Piotrowicz, Buchner, Piotrowski, & Piotrowicz, 2015)* and psychological status (*Piotrowicz, Piotrowski, & Piotrowicz, 2016*), with similar QoL improvement to standard rehabilitation (*Piotrowicz, Stepnowska, et al., 2015*). The first randomized, prospective, multicenter study (TELEREH‐HF) showed that hybrid telerehabilitation and telecare in HF patients was more effective than usual care in improving peak VO2, 6‐minute walk distance, and QoL, although not associated with reduction of 24‐month mortality and hospitalization except in the most experienced centers (*Piotrowicz, Pencina, et al., 2019, Piotrowicz, Piotrowicz, et al., 2019).*

The recent Scientific Statement from the American Association of Cardiovascular and Pulmonary Rehabilitation, the AHA, and the ACC indicates that home‐based rehabilitation using telemedicine is a promising new direction (*Thomas et al., 2019*).

### 4.3 Diabetes

Diabetes mellitus is a strong risk factor for the development of morbidity and mortality associated with a range of cardiovascular diseases. Metabolic syndrome (elevated blood glucose and insulin resistance) acts via multiple mechanisms resultant in micro‐ and macrovascular complications, development of autonomic neuropathy, diastolic dysfunction, renal failure, and AF. Important management goals are lifestyle changes [e.g., diet and activity: see later section] to prevent disease development and tight glycemic control, especially for type 1 diabetes mellitus which demands lifelong rigorous self‐monitoring (*Balakumar, Maung-U, & Jagadeesh, 2016; Donnelan et al., 2019, Goudis et al., 2015, Wang, Green, Halperin, & Piccini, 2019, Wilkinson, Zadourian, & Taub, 2019; Wingerter, Steiger, Burrows, & Estes, 2020*). mHealth modalities self‐management was recommended recently by ESC guidelines on diabetes and cardiovascular diseases to (*Cosentino et al., 2020*).

Glycemic control may reduce AF development and recurrence (*Chao et al., 2012; Chang et al., 2014; Gu et al., 2011, Otake, Suzuki, Honda, & Maruyama, 2009*).

Mobile apps can facilitate self‐management by reminding regular assessment of required parameters and medications to take and provide educational tools and motivational support. Regular transmission of blood glucose levels from patients to their physicians can be based on SMS, email, or diverse web‐based services. Bluetooth‐enabled glucose meters are frequently used (*Andres et al., 2019, Garabedian, Ross-Degnan, & Wharam, 2015*). BlueStar (Welldoc, Columbia, MD), first to receive US FDA clearance for diabetes mellitus management, comes with an app which requires a physician prescription and enables patients to titrate insulin dosing by using the proprietary insulin calculator. The Freestyle LibreLink app (Abbott Laboratories, Abbott Park, IL) reads an associated continuous glucose monitoring device and displays trends (*Fokkert et al., 2017*).

Stand‐alone diabetes management apps have recently been reviewed (*Fleming et al., 2020*). Short‐term measures, such as HbA1c, may be improved by such apps in conjunction with clinical support, but many have suboptimal usability (*Veazie et al., 2018*). Phone‐based interventions were associated with improved glycemic control as compared to standard care (*Fokkert et al., 2019, Liang et al., 2011, Pillay et al., 2015, Saffari, Ghanizadeh, & Koenig, 2014*). Efficacy for improving glycemic control in randomized controlled trials has shown mixed results (*Agarwal et al., 2019, Quinn et al., 2011*). Meta‐analyses indicate that mobile phone interventions for self‐management reduced HbA1c modestly by 0.2‐0.5% over a median of 6‐month follow‐up duration, with a greater reduction in patients with type 2 compared to type 1 diabetes *(Pal et al., 2014)*. A significant impact on clinical outcomes may affect healthcare expenditures by reducing the need for in‐person contact with healthcare providers, preventing hospital admissions, and improving prognosis. In a retrospective study, the use of mHealth technologies was associated with a 21.9% reduction in medical spending than a control group during the first year *(Whaley et al., 2019).* Key determinants to successful uptake of decision‐support apps will be their user‐friendliness and complexity and the delivery of electronic communications and feedback to the patient.

### 4.4 Hypertension

Hypertension, because of its high prevalence, provides the highest attributable risk for the development of AF (*Huxley et al., 2011*).

mHealth strategies for hypertension comprise a continuum of solutions, used by consumers or healthcare providers, and includes wireless diagnostic and clinical decision‐support tools, aiming to monitor health status and improve health outcomes. BP telemonitoring is one of the most commonly used strategies and includes remote data transmission of BP and clinical information from patients in their home or from a community setting to a central service, where they are reviewed by a managing physician for treatment adjustments. Several clinical trials have shown that BP telemonitoring might be more effective than usual care in achieving target BP (*Bosworth et al., 2011, Kim, Shin, Park, & Lee, 2015, McManus et al., 2010*). A meta‐analysis showed that, compared with usual care, BP telemonitoring improved office systolic BP and diastolic BP by 3.99 mm Hg (95% confidence interval (CI): 5.06–2.93; P<0.001) and 1.99 mm Hg (95% CI: −2.60 to −1.39; P<0.001), respectively *(Duan et al., 2017*). BP telemonitoring nested in a more complex intervention, including additional support, as face‐to‐face counseling, telecounseling, education, behavioral management, medication management, and adherence contracts, led to additional and more sustainable benefit (*Duan et al., 2017; Tucker et al., 2017*)

mHealth has the potential to promote patient self‐management, as a complement to the doctor's intervention, and encourage greater participation in medical decision‐making. Indeed, the TASMINH4 unblinded randomized controlled trial showed that patients who used self‐monitoring of BP to titrate antihypertensives, with or without telemonitoring, achieved better BP control than those assigned to usual care (*McManus et al., 2018*). The self‐monitoring group that used telemonitoring achieved lower BP quicker than the self‐monitoring group not receiving telemonitoring support, but readings were not significantly different at 1 year of follow‐up. Cost‐effectiveness analysis suggests that self‐monitoring in this context is cost‐effective by NICE criteria, that is, costing well under £20,000 per QALY (*Monahan et al., 2019*).

Although mHealth options may aid hypertension management, technological barriers, high costs, heterogeneity of solutions and technologies, and lack of standards challenge clinical implementation. The 2019 ESC guidelines on hypertension stress the importance of self‐monitoring and underline the potential use of smartphone‐based solutions. Nevertheless, they do not recommend the use of mobile apps as independent mean of BP measurements (*Williams et al., 2018*).

### 4.5 Disorders Including Sleep Apnea (See also Heart Failure Section 4.2.1)

Sleep disorders are widely prevalent and contribute to cardiovascular risk and arrhythmias, especially AF (*Daghlas et al., 2019, Hirshkowitz et al., 2015, Mehra et al., 2006, May et al., 2016, May, Van Wagoner, & Mehra,* 2017), (*Institute of Medicine Report: Sleep Disorders and Sleep Deprivation: An Unmet Public Health Problem), (Institute of Medicine (US) Committee on Sleep Medicine and Research//* www.ncbi.nlm.nih.gov/books/NBK19961). This maybe because sleep disturbance is intimately tied to circadian rhythms and sympatho‐vagal balances (*Burgess, Trindert, Kim, & Luke, 1997*). Standard sleep disorder diagnostics have been validated but require technical support for data acquisition and scoring. For example, polysomnography has long been considered the gold‐standard for acquisition of rich multimodal cardio‐neurorespiratory objective physiologic data to ascertain sleep architecture, total sleep time, and cardiorespiratory abnormalities and is primarily used for the diagnosis of obstructive sleep apnea. Actigraphy has the advantage of collecting objective data over days and nights to characterize sleep–wake patterning and provide measures of total sleep time, sleep efficiency, and sleep onset latency in addition to surrogate circadian measures. However, such tests are obtrusive and expensive.

- Treating sleep apnea may reduce AF burden (*Qureshi et al., 2015, Youssef et al., 2018*).

Consumer technology directed to sleep medicine may revolutionize the detection and treatment of sleep disorders. Since such apps are preinstalled on many smartphones, sleep tracking may be among the most widely applied facets of mHealth (*Khosla et al., 2018*). Applications include mobile device applications, wearable devices, embedded devices (in the individual’s sleep environment), rings *(* https://bodimetrics.com/product/circul‐sleep‐and‐fitness‐ring), integration of accessory diagnostic monitoring (e.g., oximetry, ECG monitoring), and sleep therapy adherence monitoring. Several commercially available wearable devices measure total sleep time accurately, but not more detailed parameters such as sleep efficiency and different sleep stages (*Mantua, Gravel, & Spencer, 2016*). Preliminary data suggest that wearable devices may be capable of detecting sleep apnea with good accuracy compared to gold‐standard polysomnography (*Selvaraj et al., 2014*) and transform the approach to sleep disorder screening, diagnosis, and treatment. Sleep irregularity diagnosed by 7 day wrist actigraphy was linked to risk of cardiovascular events (*Huang, Mariani, & Redline, 2020).* Preliminary studies indicated that use of wearables may permit behavior modifications that improve sleep quality (*Berryhill et al., 2020*). In this regard, mHealth applications to sleep diagnosis and treatment promise facilitation of rhythm control.

### 4.6 Lifestyle (See Figure 5)

#### 4.6.1 Physical activity

Physical activity is any bodily movement from skeletal muscle contraction to increase energy expenditure above basal level. Athletic activity varies from recreational sports to competitive events. There is a compelling evidence that regular aerobic exercise at the levels recommended by Physical Activity Guidelines Advisory Committee reduces the risk of a variety of cardiovascular conditions, including AF (*Everett et al., 2011, Mozaffarian, Furberg, Psaty, & Siscovick, 2008, Piercy et al., 2018*). However, the majority of the population is not engaged in physical activity at the recommended levels (*Piercy 2018*). Among patients with cardiovascular disease, patient activity measured automatically by ICDs correlated with survival following ICD implantation (*Kramer et al., 2015).* Fitness represents an enormous market for mobile technologies and significant opportunity to improve the health of a wide range of mHealth consumers. In 2017, over 318,000 “fitness and health” apps were available, almost double the number two years prior (*IQUIVA Institute 2017*). Many of these recreational apps monitor daily physical activity and support a healthy lifestyle by counting the number of steps daily, online training, and motivation coaching (*McConnell et al., 2018*).

- Cardiorespiratory fitness has an inverse relationship to AF burden (*Faselis et al., 2016*).
- Improvement in exercise capacity of 2 METs in overweight individuals may double freedom from AF (*Pathak et al., 2015*).

Consumer‐grade fitness technology includes individual fitness trackers that can stand alone, a fitness tracker that is coupled with a companion app, or an app that can be downloaded onto a smartphone, which then utilizes various features of the smartphone to measure activity and sleep. The accuracy of these measurements varies between different products and between measures within the same product (*Rosenberger, Buman, Haskell, McConnell, & Carstensen, 2016*). Furthermore, while step‐counting is long established, measuring the intensity of exercise is more complex. Although fitness technology has the exciting potential to increase physical activity by promoting goal setting and providing feedback, its effectiveness in motivating positive behavioral change remains unclear (*Sullivan & Lachman, 2017*).

One cautionary tale is the study by Jakicic et al., that examined the effectiveness of a lifestyle intervention with or without a fitness tracker (*Jakicic et al., 2016*). Two groups received instruction to promote physical activity and dietary restriction. Six months into the intervention, half of the participants were provided with an upper arm fitness tracker and web‐based support accompanying the device. The other half logged and tracked their activity and diet on a study website. Of note, the group that wore the tracker lost less weight than the group who did not. Moreover, changes in physical activity between the two groups were not significantly different. These results cast doubt on the effectiveness of fitness trackers in promoting greater physical activity, and thus, further data are required to assess the impact of this approach (See Section 5).

#### Competitive athletes

These are a unique category. Endurance athletes may have increased AF risk (*Abdulla & Nielson, 2009, Anderson et al., 2013*). Remote evaluation of ECG recordings may be useful in countries that perform preparticipation ECG screening (*Brunetti et al., 2014, Orchard et al., 2019*). Mobile devices and apps provide complex data which can be used as a self‐monitoring tool for managing training (*Aroganam, Manivannan, & Harrison, 2019, Li et al., 2016, Peake, Kerr, & Sullivan, 2018, Peart, Balsalobre-Fernandez, & Shaw, 2019, Seshadri et al., 2019*). Exercise load and performance level can be accessed on a regular basis by coaches as well as athletes. Training guided by daily monitoring of HRV parameters has also been proposed, but data are limited (*Coppetti et al., 2017, Dobbs et al., 2019, Singh et al., 2018*). Mobile devices provide the possibility of online real‐time monitoring during indoor and outdoor training and competitions. Monitoring of heart rate provides both information on performance and level of training but can also provide valuable information regarding heart rhythm irregularity suggestive of arrhythmias. Any kind of paroxysmal arrhythmia related to sport participation and detected by mobile devices designed merely for heart rate assessment should trigger further cardiological evaluation. Having in mind data indicating that sports participation may be associated with higher risk of development of AF mobile devices may serve as valuable screening tool for AF detection.

Importantly, mHealth solutions enable easy access to athletes’ medical data. The latter approach can be of special interest in management of athletes’ health during competitions abroad.

#### 4.6.2 Diet

In 2010, the American Heart Association promulgated “Life’s Simple 7” as a public health strategy to improve cardiovascular health with the motto: “7 Small Steps to Big Changes. It’s easy and simple. Anyone can do it. Start with one or two!” Unfortunately, research has shown that this strategy is anything but simple: virtually, no adults (<1%) are compliant with all recommendations and 42% are compliant with only 0‐2 recommendations (*Folsom et al., 2011*). Although there is ample evidence that weight loss and maintaining an ideal weight are beneficial in reducing AF burden and symptoms, compliance with this recommendation is poor; the reasons include among others, inability to track food intake (*Abed et al., 2013, Donnellan et al., 2019, Pathak et al., 2015).*

- Weight loss combined with risk factor modification is a Class 1 (B‐R) recommendation in treatment of AF (*January et al., 2019)*
- >10% weight reduction/ target BMI <27 kg/m^2^ reduces AF burden (*Pathak et al., 2015*).

There are currently many consumer‐oriented mobile phone‐based applications (apps) designed for tracking food intake, but their utility for use in carbohydrate counting is limited due their design (*El‐Gayar, Timsina, & Nawar, 2013*). Commonly, these consumer‐oriented apps require multiple steps. As an example, the user types in the food consumed and then scrolls through the search results to match with the program’s food and nutrient database. Next, after finding a matching food type, the user must estimate and enter an amount. These apps require significant user input and time burden along with high possibility of error. In addition, they are also plagued by uncertain accuracy. Recently, research has shown that nutrient calculations from leading nutrition tracking apps tended to be lower than results from using 24‐hour recall with analysis by the Nutrition Data System for Research (NDSR), a research‐level dietary analysis software (*Griffiths, Harnack, & Pereira, 2018*).

By contrast, a visual image‐based app, such as the Technology‐Assisted Dietary Assessment (TADA) system, directly addresses the aforementioned shortcomings (*Boushey, Spoden, Zhu, Delp, & Kerr, 2017, Six et al., 2010, Zhu et al., 2010*). This is in research phase. The TADA system consists of two main components: (1) A smartphone app that runs on either iPhones (iOS) or Android devices: the Mobile Food Record (mFR), and (2) Cloud‐based server that communicates with the mFR, processes, and stores the food images. Using the TADA system, a person takes a photograph of the meal they are planning to eat using their smartphone’s camera. The use of geometric models has permitted the TADA system to use a single image of a meal to estimate portion size to within 15% of the actual amount (*Fang, Liu, Zhu, Delp, & Boushey, 2015*). Hence, smartphone‐based technology such as the TADA system can facilitate tracking of food intake, which in turn can potentially help with weight management.

Despite the profusion of diet‐ and weight‐related apps, and the interest in weight loss in the community, there remains a dearth of high‐quality evidence that these apps are actually effective (*Dounavi & Tsoumani, 2019*). There remains a need for further evidence development before specific apps or other mHealth technology can be recommended or prescribed.

## 5 PATIENT SELF‐MANAGEMENT—INTEGRATED CHRONIC CARE

Generally, structured management programs inclusive of intensive patient education may improve outcomes (*Hendriks et al., 2012; Lin et al., 2014; Angaran et al., 2015*). These may be facilitated by mHealth.

### 5.1 Patient engagement

mHealth offers the opportunity to reach more patients more effectively. It may promote patient engagement through ease of access and wider dissemination to regions and communities who may not access health care through traditional modes due to cost, time, distance, embarrassment/stigma, marginalized groups, health inequities, etc. In this way, mHealth may facilitate information sharing and interaction between patients and HCPs without the need for an elaborate infrastructure (*Chow, Ariyarathna, Islam, Thiagalingam, & Redfern, 2016, Walsh, Topol, & Steinhubl, 2014*) (Figure 6). Apps may aid HCPs to explain the condition and treatment options, utilizing videos, avatars, and individualized risk scores, enabling greater patient understanding and encouraging a two‐way exchange of information to achieve a concordant decision about treatment.

#### Patients’ access to their own health data

A recent HRS statement advocates for transparent and secure access by patients to their digital data (*Slotwiner et al., 2019*). This enables active participation and appropriate self‐management. For instance, many patients with AF are interested in seeing their AF burden and physiologic data, similarly to patients with hypertension tracking their BP or patients with diabetes tracking their glucose. Recent systematic reviews of technology‐based patient‐directed interventions for cardiovascular disease suggest that engaging elements include self‐monitoring of symptoms and measurements, daily tracking of health behaviors, disease education, reminders, and interaction with HCPs (*Coorey, Neubeck, Mulley, & Redfern, 2018, Gandhi et al., 2017, Park, Beatty, Stafford, & Whooley, 2016, Pfaeffli Dale, Dobson, Whittaker, & Maddison, 2016*). In some cardiovascular conditions, self‐management (without any HCP input) improved key outcomes (*Hagglund et al., 2015, Varnfield et al., 2014*).

The model requires that patients assume responsibility and accountability for tracking conditions effectively and taking corrective measures. Possibly, this may be facilitated by data organization to present salient elements in a format comprehensible to the lay public. Active role of patients in decision‐making regarding the choice of treatment has been underlined by AF clinical guidance documents. Patients with AF are encouraged to be involved in decision‐taking through better understanding of their disease, which helps to improve communication between patients, their families, and doctors and improves patients’ adherence to prescribed therapy. Two applications in AF—one for patients and the other for healthcare providers—have been developed by CATCH ME Consortium in collaboration with European Society of Cardiology (*Kotecha et al., 2018*), but these have yet to be formally tested. In China, Guo and colleagues (*Guo, Chen, Lane, Liu, Wang, & Lip, 2017*) demonstrated that the mobile atrial fibrillation (mAFA) app, incorporating decision support, education, and patient engagement, significantly improved AF patients’ knowledge, medication adherence, quality of life, and satisfaction to anticoagulation compared to usual care.

Limitations should be recognized:

- Demands of self‐management may be excessive for even well intentioned patients required to be facile with setting up their own medical monitoring device, assessing frequency of download, interpreting and acting on data when required, and troubleshooting. These are not trivial challenges.

### 5.2 Behavioral modification

Individual health status has been found to be a strong independent predictor of mortality and cardiovascular events (*Rumsfeld et al., 2013*).

mHealth may catalyze positive behavioral change and facilitate health care. An induced healthy‐user effect was likely the basis of survival benefit among CIED patients adhering more closely to remote management (*Varma et al., 2015*). mHealth may support patients with text messaging (*Chow et al., 2015*) or mobile applications to remind patients of medication doses and times, as well as medical appointments (but synchronization with healthcare providers and/or EMR is generally lacking). The “just‐in‐time adaptive intervention” (JITAI) premise is to provide the appropriate type and amount of support to an individual at the correct time, with the ability to adjust depending on the person’s current internal and situational factors (*Nahum‐Shani et al., 2018*). mHealth technology is an ideal platform to facilitate JITAIs by providing “real‐time” personalized information, which can be utilized to inform the intervention delivered. JITAIs have been widely employed for health promotion and to support behavior change, but evidence of their efficacy is limited (*Gustafson et al., 2014, Patrick et al., 2009, Riley, Obermayer, & Jean-Mary, 2008*). Timing is integral to the perception of benefit, as is receptivity to accept and use the support (*Nahum‐Shani et al., 2015*). Bespoke, multi‐faceted mHealth tools, with motivational messages and incorporating gamification, are most engaging (*Coorey, Neubeck, Mulley, & Redfern, 2018, Gandhi et al., 2017, Park, Beatty, Stafford, & Whooley, 2016, Pfaeffli Dale, Dobson, Whittaker, & Maddison, 2016*).

Incorporation of gamification strategies (e.g., rewards, prizes, avatars, performance feedback, leader‐boards, competitions, and social connection) into mHealth promotes patient engagement and sustains healthy behaviors (*Blondon, Meyer, Lovis, & Ehrler, 2017, Cugelman, 2013, Edwards et al., 2016, Johnson et al., 2016, Sardi, Idri, & Fernandez-Aleman, 2017*). However, a recent systematic review demonstrated that only 4% (64/1680) of English‐language “top‐rated” health apps incorporated ≥1 gaming feature (*Edwards et al., 2016*). There are limited hypothesis‐generated data for these mHealth interventions, and their efficacy in this context is as yet unmeasured. Self‐regulatory behavior change techniques, such as feedback and monitoring (including self‐monitoring), comparison of behavior, rewards, incentives and threats, and social support, are the most common behavior change techniques employed in gamification apps and are frequently utilized in successful nongaming apps targeting health promotion and secondary prevention (*Conroy, Yang, & Maher, 2014*, *Direito et al., 2014, Edwards et al., 2016*). Engaging with apps involving gamification can also improve emotional well‐being through feelings of accomplishment and social connectivity (*Johnson et al., 2016*).

### 5.3 Patients as part of a community

Incorporation of a patient as part of a wider community may offer benefits. Social networking is widely used for health (*Fox 2011*). Online communities enable individuals to “meet,” share their experiences, discuss treatment, and receive and provide support from peers, patient organizations, or HCPs (*Fox, 2011, Swan, 2009, Swan, 2012*). While crowdsourcing via the Internet and social networks allows collective sharing and exchange of information from a large number of people, the integrity and accuracy of such information remains largely un‐vetted and as such may be unreliable (*Besaleva & Weaver, 2014*).

### 5.4 Maintaining patient engagement

Sustaining healthy behaviors and minimizing intervention fatigue is paramount to long‐term maintenance. Although mHealth may help to maintain motivation, available data demonstrate significant attrition with mHealth interventions targeting risk factors and chronic conditions, even when people report liking the intervention and have purchased it (*Chaudhry et al., 2010, Flores Mateo, Granado-Font, Ferre-Grau, Ferré-Grau, & Montaña‐Carreras, 2015, Fukuoka, Gay, Haskell, Arai, & Vittinghoff, 2015, Morgan et al., 2017, Owen et al., 2015, Simblett et al., 2018, Whitehead & Seaton, 2016, Endeavour Partners, 2017, Perez et al., 2019).*

A representative patient’s experience is described below:

A few years ago (2017), a friend told me about a new app that he had installed on his iPhone that would allow him to measure his heart rate through a fingertip pulse. Having an irregular heartbeat, under control through medication, I was very interested to try the new app. I thought it would provide me the opportunity to know more about myself, specifically how my heart operated under stress and at different times of day, before, during, and after physical exertion of a variety of my favorite sports and pastimes like tennis, golf, biking, and fly fishing.

At first, I was quite satisfied with the rudimentary calculations. Then, I noticed during my international business travels that the device was often down during US nighttime hours during which time I thought the ‘hosts’ were making repairs or improvements. I also noticed that there were several radically incorrect readings especially during early morning hours. It simply wasn’t performing up to the standards of more traditional monitoring devices. I found as well that the host’s increasing attempt to up‐sell to premium packages and other online health management tools became quite burdensome.

Before long, I felt almost addicted to the device and ultimately quit on it altogether. In retrospect, I believe that if I had had a proper introduction to the device by a trained medical specialist, I might have had a different expectation of this online tool, how to use it and how to interpret its data output.

Understanding the basis for health‐protective behavior is vital (*Dunton, 2018*). Many apps, including those from national heart foundations (*American Heart Association, 2019, British Heart Foundation, 2014, Canadian Heart and Stroke Foundation, 2014, National Heart Foundation of Australia, 2014*), are available to support healthy lifestyle choices, but their efficacy remains largely untested or is limited by design features (i.e., small sample sizes, selection bias, etc.). Cost, service connectivity, and credibility of information sources are important factors. However, patient engagement may be jeopardized by worries about privacy and personal data security (*Burke et al., 2015, Chow, Ariyarathna, Islam, Thiagalingam, & Redfern, 2016, Kumar et al., 2013, Steinhubl, Muse, & Topol, 2015*).

#### Continued clinic support

The level and duration of clinic support needed will likely depend on condition monitored and goals for treatment. Reduction in compulsory routine in‐clinic evaluations and reliance on continuous remote monitoring improved retention to long‐term follow‐up of patients with CIEDs (*Varma, Michalski, Stambler, Pavri, & TRUST Investigators, 2014*). In one HF trial, gain was related to the period of remote instruction. Whether this indicates that efficacy of the active program had peaked and stabilized or that it needed to be sustained is unclear (*Varma, 2020*). Ideally, a training program should be finite in time but its effects durable.

### 5.5 Digital divide

Although mHealth is highly promising in transforming health care, it can potentially exacerbate disparities in health care along sociodemographic lines.

Older people are perceived to engage less with mHealth. A 2017 Pew Research Center survey found that 92% of 18‐29 year olds and 74% of age 50‐64 year olds own a smartphone (*Smith, 2017*). However, the lack of familiarity with the technology and access to mobile devices, rather than lack of engagement *per se*, remain the principal barriers (*Coorey, Neubeck, Mulley, & Redfern, 2018, Gallagher et al., 2017, Tarakji et al., 2018*). Older users of mHealth prefer personalized information, which is clearly presented and is easy to navigate (*Neubeck et al., 2015*).

There is also disparity across the educational spectrum, with smartphone usage in 57% of the population with less than high school education and 91% of the population who graduated from college.

Smartphone use differs by income, with smartphone usage in 67% of the population with income annual ≤ $30,000 and 93% of the population with income ≥ $75,000 (*Mobile Fact Sheet, 2018*). Limited evidence from the USA suggests that, although there is some variation in the mHealth use related to ethnicity, black and Hispanic Americans are not disadvantaged (*Martin, 2012*). mHealth permits information and apps to be tailored appropriately for language, literacy levels (including “text to speech” technology), and cultural differences to promote engagement (*Coorey, Neubeck, Mulley, & Redfern, 2018, Neubeck, Cartledge, Dawkes, & Gallagher, 2017, Redfern et al., 2016*).

There is heterogeneity of mHealth availability among different countries (*Varma, 2020* *)*. Even some of the best studied and FDA and CE approved technologies described here may be currently unavailable due to regulatory or marketing rules or simply unaffordable to either individuals or healthcare systems in many other countries.

As healthcare systems leverage and incorporate smartphone‐based technology in their workflow and processes, a strategy is needed in parallel to ensure that those who do not have access to smartphone‐based technology will continue to receive appropriate high‐quality care. This critical initiative will require consensus and action among all stakeholders including HCPs, hospital systems, insurance providers, and state and federal government agencies. Thus enabled, mHealth promises improved patient outcomes in resource‐limited areas (*Bhavnani et al., 2018*).

## 6 CLINICAL TRIALS

mHealth may have particular impact on trials of heart rhythm disorders. Traditionally, clinical trials testing drugs and devices for arrhythmias utilized time‐to‐event outcomes and analyses, such as first recurrence of AF after a blanking period (*Piccini et al., 2017*). Patients randomized to the control and intervention would be monitored intermittently, either with ambulatory devices and/or in‐clinic visit. Such monitoring had limited sensitivity for recurrent arrhythmias, including symptomatic and asymptomatic episodes. Furthermore, time‐to‐first event may not accurately capture reductions in arrhythmia burden, which have also been shown to be beneficial in recent randomized trials (*Andrade et al., 2019*). While CIEDs such as pacemakers and defibrillators can be leveraged for continuous monitoring (*Varma, Stambler, & Chun, 2005*), these studies do not generalize to broader CIED‐free populations. ILRs may have a potential role, but are costly and unless used for clinical indications, difficult to justify simply for study event ascertainment.

There are a variety of free‐standing handheld ECG monitors, some of which have automated AF detection (Table 1). However, many do not have cellular or networking capability and therefore generally cannot transmit data or findings in real time. This is where smart‐ or mobile‐connected arrhythmia and pulse detection technologies have significant promise. These may enhance detection and measurement of clinical outcomes while also allowing for remote or virtual data collection without the need for site‐based study visits. Examples include remote rhythm assessment with single‐ or multilead ECGs from smartphone or smartwatch‐based technologies and automatic ascertainment of hospitalizations using smartphone‐based geofencing (*Nguyen et al., 2017*). These operational enhancements, in turn, can improve participant satisfaction, reduce cost, improve study efficiency, and facilitate or expand enrollment. An example is the ongoing Health eHeart study, a site‐free cardiovascular research study that leverages self‐reported data, data from wearable sensors, electronic health records, and other importable “big data” to enable rapid‐cycle, low‐cost interventional and observational cardiovascular research (https://www.health‐eheartstudy.org/).

### Screening

Two recent large‐scale studies highlight the potential advantages of mHealth for AF screening and treatment.

- *The Apple Heart Study*

This was a highly pragmatic, single‐arm investigational device exemption study designed to test the performance and safety of a PPG‐based irregular rhythm detection algorithm on the Apple Watch for identification of AF (*Perez et al., 2019,* Turakhia et al., 2019). The study was a siteless “bring your own device” study, such that participants needed their own compatible smartphone and watch to enroll online. All study procedures, including eligibility verification, onboarding, enrollment, and data collection, were performed via the study app, which could be downloaded from the app store. If a participant received an irregular pulse notification, then subsequent study visits were done via video conferencing to study physicians directly with the app. The study enrolled over 419,000 participants without pre‐existing AF in just an eight‐month period, in large part due to the pragmatic, virtual design, and easy accessibility (Figure 4). The algorithm was found to have a positive predictive value of simultaneous ECG‐confirmed AF of 0.84 (*Perez et al., 2019*). Only 0.5% of the enrolled population received any irregular pulse notification, but 3.2% of those age ≥ 65 years received notifications. However, only 153/450 (34%) patients had AF detected by a subsequent single ECG patches after the irregular rhythm notification was received. This may reflect the paroxysmal nature of early‐stage AF rather than explicit false positives. Because the study only administered ECG patch morning to those with irregular rhythm notification rather than then entire cohort or to negative controls, the negative predictive value was not estimated. It should be noticed that the Apple Heart Study was in a population without diagnosed AF; test performance and diagnostic yield could be considerably different in a population with known AF, and this software is not approved for use for AF surveillance in established AF.

- *The Huawei heart study*

A similar study was performed using smart device‐based (Huawei fitness band or smartwatch) PPG technology (*Guo et al., 2019*). The algorithm had been validated with over 29 485 PPG signals before commencement of the trial. More than 246,000 people downloaded the PPG screening app, of which about 187 000 individuals monitored their pulse rhythm for 7 months. AF was found in 0.23% (slightly lower than Apple Heart, possibly due to a younger and healthier enrolled cohort). Validation was achieved in 87% (PPV >90%) compared to 34% in Apple Heart. The results indicated that this was a feasible frequent continuous monitoring approach for the screening and early detection of AF in a large population.

A significant observation was that clinical decision‐support tools provided enabled management decisions, for example, almost 80% high‐risk patients were anticoagulated. Subsequent enrollment into the mAFA II trial showed significantly reduced risk of rehospitalization and clinical adverse events (*Guo et al., 2020*). These trial results encourage incorporation of such technology effectively into the AF management pathways at multiple levels, that is, screening and detection of AF, as well as early interventions to reduce stroke and other AF‐related complications.

- *Fitbit study*

Another large‐scale virtual study to identify episodes of irregular heart rhythm suggestive of AF was announced by Fitbit in May 2020 (*MobiHealthNews, 2020, Varma, 2020*).

### Point of Care

The next step beyond parameterizing safety could be to actionably guide therapy at the point of care (Figure 6). For example, patients could obtain ECGs before and after taking “pill‐in‐the‐pocket” antiarrhythmic drug therapy such as flecainide to confirm AF, ensure no QRS widening, and confirm restoration of sinus rhythm. A similar approach has been proposed for rhythm‐guided use of direct OACs in lower‐risk AF patients with infrequent episodes either spontaneously or as the result of a rhythm control intervention including drugs and ablation; a randomized trial is in development (*Passman et al., 2016*). The use of smartwatch‐guided rate control as a treatment strategy could also be tested, as this may provide a more personalized approach rather than prior randomized trials of lenient versus strict rate control that used population level rather than personalized heart rate treatment thresholds (*Van Gelder et al., 2010*).

### Questions

- *Generalizability*

This is key to application of results from trials. mHealth is widely available and often simple to apply and wear.

- Older individuals and those with low health literacy may find technologies difficult to use (5.5 Digital Divide), and this may be compounded by disease state, for example, previous stroke.
- Cost and service plans associated with smartphones and smartwatches may preclude their use in lower socioeconomic populations who are already under‐represented in clinical trials and in many geographies.

Thus, patients who volunteer in mHealth studies in the USA are more likely to be a white/non‐Hispanic, more educated, and less likely to have disease.

- *Adherence*

mHealth‐based evaluation of clinical endpoints may be confounded if adherence is low, particularly if there are no secondary means of endpoint assessments (*Guo, Vittinghoff, Olgin, Marcus, & Pletcher, 2017*). Virtual designs may be more susceptible to the loss of participant engagement. For example, if monitoring is completely reliant upon mobile health technology and there are no traditional measures or in‐person visits to assess arrhythmia, then significant missing data due to low‐adherence may become a major limitation that could imperil the validity and generalizability of the findings. For example, among the 2,161 of the 419,297 that received an irregular pulse notification in the Apple Heart Study, only 945 completed a subsequent protocoled first study visit. Of these 658 ambulatory ECG patches shipped, there were only 450 with returned and analyzable data (*Perez et al., 2019*).

Development of effective strategies to increase retention and maintain high engagement remains an unmet need and is an area ripe for more research.

- Outcomes

These are key to adoption and reimbursement. More specifically, the clinical and prognostic impact of new outcome measures based on mobile health technologies may not be clear.

This is important for AF. For example, how do changes in AF burden compare to reductions in time to symptomatic sustained AF? Should AF identified on near‐continuous smartwatch monitoring be considered equivalent to AF diagnosed at hospitalization or in clinic? There is a growing body of literature that the “dose” of AF burden matters for a variety of important clinical endpoints, including stroke, HF, and death (See Section 3.1.3) (*Chen et al., 2018, Glotzer et al., 2009, Kaplan et al., 2019, Piccini et al., 2019, Wong et al., 2018*). Does pill‐in‐the‐pocket DOAC treatment of PAF adequately cover the risk of stroke? Some measures remain less well studied, like the occurrence of irregularity with a wearable pulse‐based monitor system, particularly without ECG confirmation.

Since these mHealth prediagnostic or diagnostic tools may then be directly tied to initiation or termination of treatment, rigorous evaluation of clinical safety and efficacy will be required and, in some cases, warrant a combined drug‐device regulatory approval.

Despite these challenges, there is enormous potential for patients to use these technologies to self‐monitor their arrhythmia treatment and extend this to manage comorbidities (See Section 4). The process of data transparency and accessibility to the patient may improve the patient’s engagement with their overall care, even if the data are not directly actionable by the patient. The restrictions to clinic access during the SARS‐Cov‐2 pandemic have accelerated the adoption of mHealth solutions (*Varma et al., 2020*). ECGs for clinical trials were recorded by smart devices and assessed at virtual visits instead of routine in‐person evaluations. In some cases, the entire management of clinical trials went online.

## 7 OPERATIONAL CHALLENGES

### 7.1 Healthcare System—Ehealth Monitoring and Hospital Ecosystem

#### Transmission

A fundamental but as yet unresolved challenge of incorporating mHealth into clinical practice is the channel of data communication between patient and provider. This may differ depending upon whether the data are physician‐facing (e.g., for CIEDs) or patient‐facing (consumer digital health products, e.g., the Apple Watch (Apple Inc., Cupertino, CA)).

*CIEDs:* Experience with CIEDs provides a framework. CIEDs generate voluminous quantities of eHealth data. In a single patient, this may be generated from distinct sources, that is, remote monitoring and in‐person interrogations. Transmission from remote monitoring has been well worked out: data flow from the CIED to the remote transceiver and then to the manufacturer’s server for access by individual practices. Unfortunately, this is usually retrieved in an image format rendering the granular data uninterpretable by the practice’s electronic health record (EHR). When shared with the patient, the image file is posted on the EHR’s patient portal. These files are difficult for physicians to interpret and practically uninterpretable by the lay public. In order to engage patients and caregivers, the data will need to be provided in a format that enables the lay public to get a high‐level summary of key features (such as battery status and remote monitor function status) with explanations and the ability to drill down to the more granular details for those individuals who wish to do so.

*Consumer digital health product data:* Consumers are rapidly adopting products to monitor their health status for early detection of abnormalities as well as for managing chronic diseases. These tools empower and engage patients in managing their health, but the very basic task of sharing the data with their healthcare provider presents challenges. From a technical standpoint, many EHR portals do not permit patients to send attachments. Therefore, the patient and provider are left using email, which is not considered secure or HIPPA or GDPR compliant. Even if the EHR portal accepts attachments, incorporating the digital health data into the EHR remains ad hoc and inconsistent. The logistical and practical concerns frighten many care providers into discouraging their patients from using these devices. Concerns among providers include the fear of being inundated with unnecessary transmissions to review as well as the concern that patients may send inappropriate data, for example, BP or glucose monitoring data to their electrophysiologist. Cloud‐based storage may avoid some of these challenges.

#### Interoperability—Lack of organized infrastructure to receive incoming the data

Assimilating the data obtained from digital health tools, whether implantable or wearable, is proving to be one of the greatest clinical challenges. Clinicians feel increasingly burdened as both the volume of data as well as the sources of data increase. Creating the nomenclature and data models that would enable the information to be incorporated in the electronic medical record is less a technical challenge, but more a political challenge. It requires a consensus from the clinical community regarding definitions of the terminology and agreement on what data are required. For example, for pacemakers, there must be agreement on the definition of battery longevity, pacing thresholds, mode switch, etc. For CIEDs, this work has been done (https://www.iso.org/standard/63904.html , *Slotwiner et al., 2019*). The next step is for EHR vendors to support the agreed‐upon nomenclature and the data standard in which it is communicated. With these 2 building blocks, digital health data can be assimilated into the clinical workflow, enabling healthcare providers to review, manage, and document clinical impressions and recommendations within the environment of their EHR. This work is ongoing in the domain of CIEDs but has not started for wearable devices. It requires a coalition of clinicians, engineers, regulatory agencies as well as regulatory and/or financial incentives for vendors. A high‐efficient computerized system with huge storage is necessary infrastructure and may provide the platform for predictive analytics.

#### Interoperability—Lack of organized infrastructure to transmit data and instructions

There is interest in mHealth to support patients with text messaging (*Chow et al., 2015*) or mobile applications to remind patients of medication doses and times or medical appointments. To be effective, this requires synchronization with healthcare providers, ideally by integration with the EMR, allowing changes in medications and doses, as well as appointments, to flow between patients and clinicians in an accurate and bidirectional manner (*Spaulding et al., 2019*). However, EMR systems software is lacking such functionality and interoperability at this point (*Ratwani et al., 2018*).

### 7.2 Cybersecurity guidance for mHealth devices

Interconnection of medical devices and clinical data promises facilitation of clinical care but also creates opportunities for intrusions by maleficent actors (i.e., hackers) to disable systems and/or access private health information (PHI) (*Jalali, Russell, Razak, & Gordon, 2019, Kruse, Frederick, Jacobson, & Monticone, 2017*). The motivation is largely financial. Healthcare facilities and medical device companies present attractive targets because a number of attack strategies can yield large financial rewards:

1. Ransomware. A hospital’s systems can be locked out (e.g., data may be encrypted) until the attacker is paid *(Mansfield-Devine, 2016, Network Security 2016*)
2. Theft and sale of patient data (i.e., PHI).
3. Company attack. A hacker may identify flaws in a system or device, short the company’s stock, and then make the flaws public. Alternatively, a maleficent user may try to harvest insider information from a breached company’s network. Attackers may compromise a company, but not take any of the above actions. Instead, they may sell their methods or credentials to another group who will use them (*Perakslis, 2014*)

Scenarios where a cyber attack results in the deaths of individuals or groups (e.g., by corrupting the firmware of a pacemaker or insulin pump) can be easily imagined and have been demonstrated by researchers (*Klonoff, 2015*), but to date, no such attack is known to have occurred in the real world. It is possible that that this is because attacks against organizations yield greater gain than attacks against individuals.

It is essential therefore to establish best practice methods to maintain patient safety and privacy in this new ecosystem of remotely managed devices and mass data collection.

#### 7.2.1 Hacking strategies and methods in mHealth technologies

Often times, attackers will not directly compromise the system that they are after; they will instead start by compromising a weaker link. For example, if the goal is to obtain PHI about a specific patient, they may attempt to get the patient (or a staff member) to install a malicious app, compromising the rest of the phone, including email and other credentials. From this point, the attacker is in a better position to attack the actual target. The process of chaining exploits to work through a system is called *pivoting*. Each pivot or “hop” enables new privileges that bring the hacker closer to desired goals.

The easiest thing to exploit is often a person with *phishing* campaigns. A compromised email account can be used to reset passwords for other services and to distribute more realistic phishing messages. More technical attack pathways are used to compromise the remote‐monitoring components of a healthcare system, for example, wireless links (bluetooth, wifi, etc.), Internet and local network communications or servers (databases, web frontends, file servers, etc.)

#### 7.2.2 Recommendations to the manufacturer

It is not possible to create systems that cannot be hacked. However, systems/devices should be designed to *fail gracefully* in conjunction with a plan. This enables rapid correction in the event of intrusion.

Business decisions (e.g., budget, timeline) should not override security which should be the priority. Attempting to close or obscure devices/protocols is not a solution, and the so called *security through obscurity*, as a defensive measure, has long been rejected as inadequate (*Shanon, 1949*). A balance between usability and security has to be struck carefully. Securing devices against attackers, while keeping them open to clinicians is a difficult task. In mHealth, this difficulty can be amplified by the dependence on the patient’s devices (e.g., smartphone) and practices, which are outside the control of a healthcare IT system. An example of an engineering compromise in implantable cardiac devices is the requirement for important wireless communications to only work at very short ranges. These communications could be made more secure but less usable (e.g., requiring wires), or less secure but more usable (e.g., using Bluetooth).

#### 7.2.3 Recommendations to clinicians and administrators

The organization should be designed with *security in layers* (also called *defense in depth)*, where each system is protected with more than one layer of security. Hence, a breach in one layer will not necessarily result in total compromise. For example, a database may 1) require a password, 2) only grant a minimum level of access to each user, and 3) only accept internal connections. Thus, if a user’s password is compromised (#1 failed), an attacker still cannot use it remotely. If the server is accidentally opened to remote access (#3 failed), the attacker can still only access that one user’s data. Other innovative solutions include delegating security to a personal base station to use a novel radio design that can act as a jammer‐cum‐receiver (Gollakotta et al., 2011).

When recommending devices for patients, it is important to consider the potential privacy/security weaknesses compared to alternatives, ensure the patient is informed about these tradeoffs, and review how the manufacturer has responded to security incidents in the past (*Saxon, Varma, Epstein, Ganz, & Epstein, 2018*). However, the lack of outcome data, combined with the lack of documented real‐world instances of actual cybersecurity intrusions to these devices or to peripheral products that support device connectivity (programmer, home communicator, database, communication protocols), pose a difficult risk–benefit assessment for clinicians and patients alike.

Regulatory frameworks around cybersecurity are changing rapidly (*Voelker, 2018*). The FDA (as well as other regulatory agencies worldwide) now includes security as a part of device safety/efficacy checks, and we encourage readers to report security issues to manufacturers and the government (e.g., through FDA Medwatch) (*Shuren, Patel, & Gottlieb, 2018*).

#### 7.2.4 Recommendations to patients

Clear advice to patients concerning cybersecurity should be followed by a formal patient informed consent.

### 7.3 Reimbursement

Reimbursement is a powerful driver of adoption of new clinical pathways and typically instituted once an intervention has been proven scientifically valid and cost‐effective (*Treskes, van der Velde, Barendse, & Bruining, 2016*). This process has only just started in mHealth and may be more complex to measure given the wide scope of telemedicine.

- *Reduced costs*

This technology may promote an effective means for early diagnosis and treatment of arrhythmias and associated comorbidities, leading to benefits of screening, prevention, and early treatment, thereby reducing adverse effects related to delayed therapy and utilization of costly healthcare resources (e.g., ER visits or hospitalizations). mHealth may help individuals adhere to health recommendations, empower active participation in lifestyle changes to modify cardiovascular risk profile, and promote adherence to medical therapy (*Feldman et al., 2018*). Together, these may reduce the burden of chronic disease and associated long‐term disability. However, assessment of these longer‐term cost advantages is challenging, and value will vary according to country and healthcare system.

- *Increased costs*

Conversely, there are costs associated with administering mHealth programs. The widespread availability of smartphones and other commercially available mobile devices will generate a significant amount of inconclusive or false positive findings, which will in turn lead to additional testing for validation, thereby increasing utilization of healthcare resources. Widespread implementation of screening programs would require additional consideration of costs related to detection of arrhythmias in currently unscreened populations. Healthcare providers will also be required to spend time reviewing and interpreting potentially voluminous results (and associated phone calls) prior to making additional evaluation and management decisions. This requires financial compensation in order to maintain a viable practice.

- *Remote monitoring of implanted devices*

This provides valuable experience. RCTs conducted over many years that demonstrated safe and effective replacement of traditional in‐clinic evaluations, and more effective discovery of asymptomatic clinical events (*Varma, Epstein, Irimpen, Schweikert, Love, & TRUST Investigators, 2010*). Health‐economic studies like EuroEco (ICD patients) showed that clinic time needed for checking web‐based information, telephone contacts, and in‐clinic discussion when required was balanced by fewer planned in‐office visits with remote monitoring, resulting in a similar cost for hospitals vs. purely in‐office follow‐up (*Heidbuchel et al., 2014*). From a payer perspective, there was a trend for cost‐saving given fewer and shorter hospitalizations, seen also in other trials (*Crossley et al., 2011, Guedon‐Moreau et al., 2014, Hindricks et al., 2014, Mabo et al., 2012*). However, in systems with fee‐for‐service reimbursement, less in‐office visits (and hospitalizations) will lead to less income for the providers (i.e., physicians and hospitals) without adaption of the new remote‐monitoring paradigm. This illustrates the complexities in reimbursement.

Currently, remote‐monitoring reimbursement (e.g., USA, Germany, France, UK) is implemented in a discrete way following the protocols of randomized trials like TRUST or IN‐TIME (*Hindricks et al., 2014, Varma, Epstein, Irimpen, Schweikert, Love, & TRUST Investigators, 2010, Varma, Michalski, Epstein, & Schweikert, 2010*), with billing after demonstration of a remote contact, with a maximum number per year. Given the technological trend toward more continuous transmissions, and decision‐support server systems that alert healthcare providers of potentially relevant information, possibly a subscription‐based system providing a lump sum per year per followed patient may be more effective. This should cover costs of hardware, software, and other services (like potential use of third‐party data monitoring centers) and would result in a much better prospective budgeting for both healthcare insurers and providers. This scheme may be apt for mobile technology.

It is anticipated that mobile health technology may provide a more efficient and cost‐effective approach to healthcare delivery that could improve clinical workflow and enhance clinical care when integrated into clinical practice (*Jiang, Ming, & You, 2019*). Linking this to improved outcome will be an important driver of reimbursement, for example, for a process leading to an arrhythmia management decision (but not when monitoring the large asymptomatic population without risk factors). Ongoing studies evaluating mobile technology, such as use of a smartphone ECG for AF screening in the AF SMART II (Atrial Fibrillation Screen, Management and Guideline Recommended Therapy) study, include a cost‐effectiveness analysis (*Orchard 2018*). Responsibilities for reimbursement may extend beyond traditional parties in health care and drive novel pathways. Mobile device companies are clearly interested in reimbursement issues, evidenced by contact between Apple health executives and insurance companies *(Bruining et al., 2014*). Initiatives undertaken in the USA are described in Appendix.

### 7.4 Regulatory landscape for mHealth devices

The pace of changes and improvement of digital technology is furiously fast. With the release and spread of the 5G cellular technology, this growth will probably be strengthened, and new frontiers around data streaming and associated analytics will be crossed. Unfortunately, this growth has been slower in the field of digital technologies, particularly in the United States. The reasons are probably linked to the unique relationship between the government and its healthcare system. In the United States, mHealth technologies are primarily led by private organizations operating under constraints linked to financial incentives (CMS reimbursement guidelines), patient privacy (Health Insurance Portability and Accountability Act), and patient safety (Food and Drug Administration, FDA). These constraints have become obsolete with the development of the digital health technologies and novel mHealth devices, and a new regulatory paradigm is being formed.

The FDA released an entirely new section under the Medical Device category called “Digital Health” which is managed by the Center for Devices and Radiological Health (CDRH) (*Shuren, Patel, & Gottlieb, 2018, Center for Devices, Radiological Health, 2019*). This development was triggered and supported by the 21st Century Cures Act signed into law on December 13, 2016. It is designed to help accelerate medical product development and bring new innovations and advances to patients. The FDA Digital Health policy is currently defined under three main categories: General Wellness, Mobile Medical Apps (MMAs), and Clinical Decision‐Support Systems. mHealth devices are present in these three categories which are defined as follows:

A wellness device is developed “for maintaining or encouraging a healthy lifestyle and is unrelated to the diagnosis, cure, mitigation, prevention, or treatment of a disease or condition” (21 CCA Section 3060 (a)(o)(1)(B)). The FDA‐regulated MMAs on the other hand as software that is focusing on traditionally regulated health functionalities and is categorized as software as a medical device (SaMD). The SaMD must be developed under well‐defined frameworks involving specific software development life cycles (IEC‐62304), risk assessment, reliability demonstration, and safety that includes cybersecurity. The clinical decision‐support (CDS) systems may rely on mHeath devices, or be included in mHeath devices. The definitions of a CDS are provided in the 21 CCA, Section 520 (o)(1)(E). Briefly, they involve the presentation of medical data, recommendations to physicians about the prevention, diagnosis, or treatment of a condition or disease. It is not the intent that the healthcare professional primarily relies on this information to make a clinical diagnosis or treatment decisions. If wellness devices do not require FDA approval to be commercialized both SaMD and CDS do.

The regulatory policies are changing and adapting over time to fit the technology development of mHeath devices. Today, the time required for approving new technologies is significantly longer than the pace of change of the mHealth technologies. Hence, streamlining the regulatory submission process is of great interest to many stakeholders. One of the very recent initiatives in the USA designed to address this challenge is the FDA’s digital health software Precertification program (Pre‐CERT) (*Lee & Kesselheim, 2018*). The Pre‐CERT is developed to shift the current paradigm of SaMD submission. The program is ambitious and proposes to expedite regulatory review for the companies that can demonstrate a series of components that includes process certification, postmarket review, and real‐world evidence (among others). It is expected that a company gaining FDA Pre‐CERT could ultimately eliminate or streamline their regulatory submission process depending on the risk associated with their SaMD technologies. Started in 2019, this initiative currently involves international companies that are pushing their wellness technologies into the clinical realm. This type of new regulatory framework will certainly help corporate America to accelerate the commercialization of their products, but the Pre‐CERT might be much more difficult to reach by smaller companies that do not have the resources to demonstrate the level of trust, and to implement the level of verification and transparency Pre‐CERT requires.

## 8 PREDICTIVE ANALYTICS

Artificial Intelligence (AI) is a broad term that describes any computational programs that normally require human intelligence such as image perception, pattern recognition, inference, or prediction (www.oed.com *; Kagiyama, Shrestha, Farjo, & Sengupta, 2019*). Most commonly, AI is implemented using analytical methods of machine learning or deep learning. These methods are well suited for pattern classifications, such as images, including ECG.

The potential synergy between AI and mHealth has excited the healthcare community since this may enable solutions to improve patient outcomes and increase efficiency with reduced costs in health care (*Davenport & Kalakota, 2019; Marcolino et al., 2018*). Smartphone apps and wearable devices generate a huge amount of data that exceed the human capacity of integration and interpretation (Steinhubl, Muse, & Topol, *2015*). Biometric datasets of astronomical proportions may be compiled. This knowledge may be directed to treat an individual or understand populations. For instance, 6 billion nights of surrogate sleep data reflecting global sleep deprivation may potentially inform public health initiatives (Pogue, 2020; https://aasmorg/fitbit‐scientists‐reveal‐results‐analysis‐6‐billion‐nights‐sleep‐data). Mobile health with Internet connection enables cloud‐based predictive analytics from individual‐level information (*Bumgarner et al., 2018, Nascimento et al., 2018, Ribeiro, Paixão, et al., 2019*).

Cardiology has been an early area of investigation in AI due to the abundance of data well suited for classification and prediction (*Seetharam, Kagiyama, & Sengupta, 2019*). Neural networks have been tested, trained, and successfully validated to be at least as accurate, if not more, than physicians in diagnosis or classification of 12‐lead ECGs and recognition of arrhythmias in rhythm strips and ambulatory ECG recordings (*Hannun et al., 2019, Ribeiro, Ribeiro, et al., 2019, Smith et al., 2019)*. They have also been shown to successfully estimate ejection fraction, identify left ventricular dysfunction, and even diagnosis diagnose diseases such as hypertrophic cardiomyopathy from the echocardiogram (*Zhang et al., 2018*). More recently, neural networks have also aided in gathering new dimensions of information, such as identifying left ventricular dysfunction (*Attia, Kapa, et al., 2019*). These methods have the potential for a point‐of‐use diagnosis of a wearable sensor or consumer device and without delays of requiring clinical conformation, although rigorous safety assessments of unsupervised use will be necessary. More recently, AI methods have also been applied to prediction, not just classification, for example, using 12‐lead ECG to predict risk of AF from a sinus rhythm ECG (*Attia, Noseworthy, et al., 2019b).*

Already, AI has been embedded in mHealth applications, such as smartwatch and smartphone‐connect ECG semi‐automated diagnosis of arrhythmias (*Bumgarner et al., 2018, Halcox et al., 2017*). These diagnoses are intended to serve as prediagnostics rather than supplanting a physician interpretation. Application of artificial intelligence techniques to point‐of‐care ultrasound in the development of machine‐learning systems may aid in the optimization of acquisition and interpretation of a high volume of images, reduce variability, and improve diagnostic accuracy (*Chamsi‐Pasha, Sengupta, & Zoghbi, 2017*). AI‐based prediction models have been developed for HF and AF, although sometimes the accuracy of the AI‐derived models seems to be rather limited or not superior than those derived from conventional methods (*Awan, Bennamoun, Sohel, Sanfilippo, & Dwivedi, 2019, Clifton, Niehaus, Charlton, & Colopy, 2015, Frizzel et al., 2017, Goto et al., 2019, Safavi et al., 2019, Tripoliti et al., 2019*). mHealth specific investigations are few. Results from the LINK‐HF study were encouraging. A cloud‐based analytics platform used a general machine‐learning method of similarity‐based modeling which models the behavior of complex systems (e.g., aircraft engines) to create a predictive algorithm for HF decompensation, using data streamed from a chest patch sensor.

Several limitations should be considered and roadblocks removed before AI‐based mHealth strategies become routinely incorporated in clinical practice (*Kagiyama, Shrestha, Farjo, & Sengupta, 2019, Powell, 2019, Ribeiro & Oliveira, 2019, Steinhubl, Muse, & Topol, 2015*). Studies on AI are still scarce and based on observational studies and secondary datasets. Validation in other clinical settings and a deeper evaluation of their meaning in every day practice are generally lacking. Thus, high‐quality evidence that supports the adoption of many new technologies is not available. Most algorithms work with the "black box" principle, without allowing the user to know the reasons why a diagnosis or recommendation was generated, which can be a problem, especially if the algorithms were designed for a different environment than the one that the current patient is inserted (*Ribeiro, Ribeiro, et al., 2019, Weng, 2017*). Issues regarding cost‐effectiveness, implementation, ethics, privacy, and safety are still unsolved.

## 9 FUTURE DIRECTIONS

**Figure 7:**
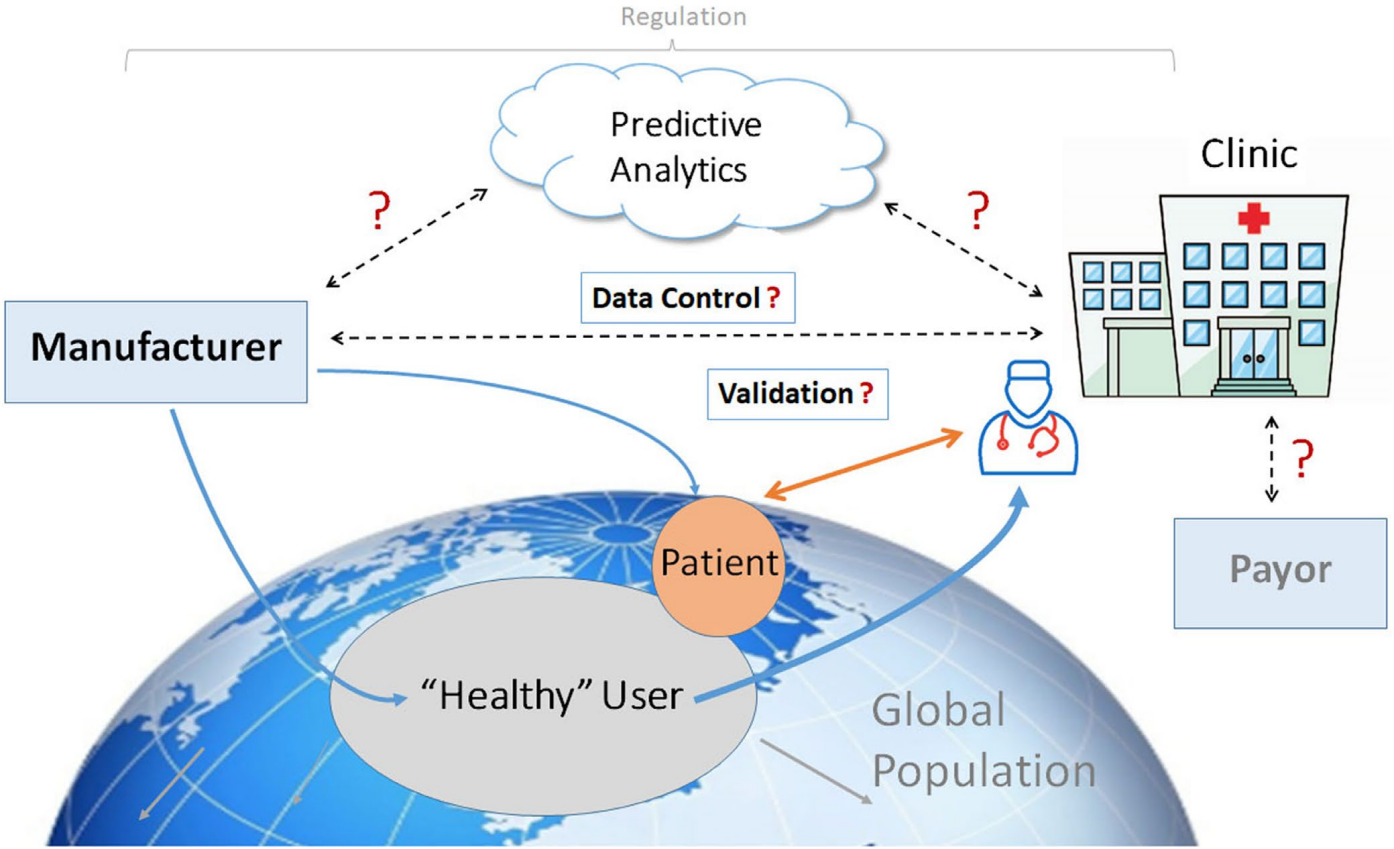
Connectivity and Questions. Multiple levels of cooperation among a variety of stakeholders are needed to capitalize fully on the vast potential of mHealth, but many questions remain unanswered. Healthy consumers (increasing) predominate among mhealth users. Only a minority of patients are prescribed these digital tools. Potential health benefits of mHealth may be realized when manufacturer participates with clinic for validation in defined disease states. Parties responsible for data control—and therby predictive analytics—need to be defined. Ultimately, the payor and physician need to be convinced of benefits before digital tools are firmly embedded in clinical practice.

mHealth is disruptive at multiple levels of health care but requires significant investment in validation, demonstration of clinical utility and value. Stakeholders, each with independent concerns and constraints, (Table 5) lack consensus or coordination with design, use cases, and implementation (Figure 7). Thus, formal recommendations for integration of mHealth into clinical practice cannot be made at this time. This is exemplified by the US Preventative Services Task Forces statement that “*evidence is insufficient* to initiate therapy for AF detected by mHealth”—despite the fact that AF has been an early use case with strong patient and clinician interest (*Curry et al., 2018*). Thus, mHealth devices are currently nonprescription devices marketed directly to consumers to track data without enabling interventions.

**Table 5:** Conditions, stakeholders and expectations

	Applications/ Conditions	Opportunities	Challenges to resolve
Bio‐signals monitored	Diverse	Multiparametric trending Contactless screening	Lack of validation Transmission frequency Ethics
Target condition	Arrhythmias Treatment Follow‐upRehabilitation Lifestyle modification Chronic disease	Screening Prevention Facilitate management	Lack of outcome data
Users	Healthy consumers	Increase use by patients	Managing the “worried well”
Patient Expectations	Confidence Engagement Education	Data access Real‐time treatment Self‐management	Data access Driven by popular press Excessive focus on data without clinical context Digital divide Lack of Internet access
Physician Expectations	Versatility	Validation Improve patient outcome Reduce in‐clinic visits Real‐time patient treatment Predictive Analytics Precision Medicine	Absence of FDA approval Lack of outcome data Establish transmission frequency Define clinical actionability Manage false positives Standardize data flowManage data overload Interoperability with EMR Mechanism for feedback to patients for treatment decisions; Assurance of patient adherence Physician or Manufacturer? Reimbursement Legal responsibility
Hospital	Improve efficiencies Improve access	Predictive analytics Interoperability Cybersecurity Reimbursement	Lack of outcome data Value impact Legal responsibility
Technology/ Manufacturer	Direct to Consumer Sales	Patient care Community care	Learn treatment pathways Partner with clinic Legal responsibility Predictive analytics
Payor	Reduce costs Improve outcome	Cost–benefit analysis	

Some of the steps needed to standardize mHealth applications are outlined below.

**1. Validation**

- Promote standards and create tools for the comparative assessment of functionality, relative to a medical use device.

Results from different devices applied to the same condition may not match: for example, the diagnosis of AF by ECG or PPG based systems are made very differently. This has significant implications for medical decisions.

**2. Identify clinical care pathways**

- *Screening*
  - Assess value according to the population addressed
  - Establish a uniform set of criteria for clinical actionability (*Slotwiner et al., 2019*).

Screening should be medically directed and not driven by commercial interests. Caution should be exercised in extrapolating management strategies learned from cohorts with clinically diagnosed AF (usually from healthcare system data, trials or inpatient registries) to AF detected with mHealth technologies (“healthy consumers”). Data from low‐risk populations carry a relatively high risk of false positives, which may generate additional tests with resultant clinical risk to patient (even inducing anxiety rather than reassurance), risk from overtreatment, and costs to the payor. There is a risk that unless directed to a higher risk population, screening for AF using mHealth technologies may fail and follow the trajectory of many medical screening programs throughout history.

*Key knowledge gap*—Identify characteristics (duration, episode number/ density) and risk factors that justify anticoagulation for mHealth detected AF.

- *Disease management*
  - Identify conditions and schedules for home‐based therapeutic strategies that may reduce dependency on clinic evaluations (as shown for CIEDs)
  - Identify signals that predict decompensation and design pre‐emptive interventions
  - Assess efficacy of therapies.
- *Outcomes*

Evidence for benefit of mHealth directed:

- Arrhythmia treatment
- Management of modulating factors (e.g., comorbidities, lifestyle modifications).

**3. Implementation**

- *Cost‐effectiveness*

Eg impact of improved clinical workflow and enhance clinical care, according to condition (*Jiang, Ming, & You, 2019*).

Impact on healthcare system and reimbursement

Impact on costs to patient or consumer.

- *Public health and Professional society initiatives*

Education, awareness

Bring together stakeholders

Guidelines.

**4. Patient Self‐management**

Patients control the intensity of monitoring and act on patient‐facing data. Frequency of data acquisition is sporadic determined by, for example, convenience, or following symptoms, or recreational. This strategy is likely insensitive for events and rarely delivers rapid clinical actionability for life‐threatening conditions. What is required is as follows:

- Education on which data are clinically actionable in individual’s clinical context and
- Tailor monitoring schedule accordingly
- Proof of safety.

In one recent example illustrates an on‐demand use. The Fibricheck app was utilized by patients to monitor rate and rhythm for a week prior to teleconsultations during the COVID‐19 pandemic to enable remote assessment of the disease state and support treatment decisions. This was regulated by a time‐limited prescription to use the app for a predefined period, avoiding unnecessary data‐load and additional follow‐up patients‐contacts (*Pluymaekers et al., 2020*).

- Patients’ legal right to their medical data to include data collected from nonmedical (i.e., consumer) products.

**5. Manufacturer**

mHealth introduces the manufacturer as a party with significant responsibilities. mHealth tools largely have been developed as consumer‐facing technologies accessible to a broader market through retail channels rather than through established medical supply channels. This may make business sense for the technology supplier, given high community penetration of wearable, smart‐technology devices (1 in 10 Americans (30 million total)). However, a direct to consumer healthcare delivery bypasses both the clinician, healthcare system, and insurer, without addressing the needs of health professionals—who remain responsible for clinical decision‐making on acquired data. Any advance toward medical application (beyond toys for the worried well/ wealthy well) will require manufacturers to:

- Facilitate accessibility and affordability
- Engage with clinicians to engineer devices according to clinical needs and partner in validation. This is vital, since physician carries ultimate responsibility for medical decisions and is best positioned to guide development and application
- Define role as data controllers (e.g., GDPR in Europe).

**6. Assign responsibilities**

- Identify parties (manufacturer, hospital, third party) responsible for cybersecurity, data protection, and liability for mis‐diagnosis or missed diagnosis
- Define standard of care for clinic response time according to condition.

This assumes greater significance as clinical decisions become enabled in real time using cloud processing resources linked to enhanced data transmission rates (5G) and Internet of Things (IoT) and scalability increases.

- Ethical and societal issues with multiple screening (*Yan et al., 2020, Turakhia, 2020*).

**7. Healthcare Delivery**

Interconnectedness between individual applications and with existing healthcare architectures may reshape the current environment.

- “Exception‐based” ambulatory care, that is, see patients as they need to be seen
- Centralized (cloud) based processing to forward only clinically relevant data to physician/clinic.
- Identify at‐risk patients early (even before symptoms develop) and permit pre‐emptive care (*Boehmer 2017, Rosier et al., 2016*).
- Pooled population screening—altering the paradigm of individual screening (*Yan et al., 2019, Turakhia, 2020*)
- Extend the role of wearables from ambulatory to in‐hospital care, for example, replace traditional wired monitoring of single parameters for individual analysis, to wireless monitoring of multiple parameters.

For example, a waterproof ring technology (Bodimetrics) was used for multiparametric monitoring (heart rate, sleep, oxygen desaturation index, steps, and calories burned) in ICU management for COVID 19 patients. The ring links to a smartphone or centralized hub in hospitals and permits data sharing and cooperative treatment (https://bodimetrics.com/product/circul‐sleep‐and‐fitness‐ring/).

- Extend function from monitoring only to intervention
- Enable remote programing of therapeutic implantable devices.

For example, CIEDs, emerging wearable cardioverter‐defibrillators, are incorporating smartphone Bluetooth® Low Energy (BLE) based connectivity for the transmission, display, and interpretation of transmitted data by patients and their clinicians. This may permit reprograming of parameters like diagnostic data, detection zones, clearing counters, AV delays/PVARP adjustment, upper rate and lower rate adjustments, reprogram amplitude adjustments; MRI mode, and enable emergency therapies or disable inappropriate therapies due to lead fracture / incessant SVT/ double counting.

- Enable interventional procedures, for example, Tele‐Robotic ablations models which could improve access to patients living in remote areas with highly skilled EPs operating remotely (*Choi, Oskouian, & Tubbs, 2018, Haidegger, Sándor, & Benyó, 2011, Shinoda et al., 2020*).
- Enable precision medicine by incorporation of the wider range of mobile signals seamlessly into genetic and clinical profile, with environmental and lifestyle data (“big data”). (https://ghr.nlm.nih.gov/primer/precisionmedicine/initiative).

## CONCLUDING REMARKS

mHealth application is at different stages of evolution around the world. Few of the technologies described are universally approved and/or affordable in all countries. As a result, this document reflects largely US perspectives. The experience described may serve to guide other members of the international professional bodies endorsing this collaborative statement. The World Health Organization envisioned that increasing the capacity to implement and scale up cost‐effective innovative digital health could play a major role in toward achieving universal health coverage and ensuring access to quality health services, at the same time recognizing barriers to implementation similar to those discussed in this document. Some of these can be resolved rapidly, as seen in response to the recent SARS‐CoV‐2 global pandemic that forced a need for contactless monitoring and thereby adoption of digital tools (*DHSS, 2020, FDA, 2020, Varma et al., 2020*). Regulatory bodies were responsive, approving technologies, relaxing rules confining use of telehealth services within borders and to certain patient populations, and creating a reimbursement structure, illustrating that appropriate solutions can be created when necessary.

Demonstration of the clinical utility of mHealth has the potential to revolutionize how populations interact with health services, worldwide.

## Supporting information

Supplementary Material

## Appendix 1

In the United States, reimbursement for medical services is guided primarily by the Centers for Medicare & Medicaid Services (CMS). The American Medical Association’s Current Procedural Terminology (CPT) Committee develops descriptive codes for each medical service and assigns a CPT code. Each CPT code is then referred to the association’s Relative Value Update Committee to develop a recommended relative value unit (RVU) which determines reimbursement. CMS usually accepts the recommendations from the AMA. Presently, the CPT Committee is developing codes to represent the clinical work involved in managing mHealth data. These codes will then be evaluated and assigned RVU values. If accepted by CMS, these will be included in the Medicare Fee Schedule and go into clinical use. This process typically takes 2 years. Once the codes and services are approved by CMS and published in the Fee Schedule, other insurers typically accept them as well (at the time of writing CPT 99091 and CPT 99457 had received approval).

In 2015, the CMS in the USA initiated a new chronic care management code that reimburses primary care practices for nonface‐to‐face care for chronic care management (CCM) payment. In November 2018, CMS finalized plans to reimburse healthcare providers for certain remote patient monitoring and telehealth services. These changes focused on three new CPT codes that separate remote patient management (RPM) services from telehealth services. The new CPT codes include #99453, 99454, and 99457. The first two codes describe remote monitoring of physiologic parameters, but do not specifically include ECG monitoring. The third code provides management services, 20 minutes or more of clinical staff/physician/other qualified HCP time in a calendar month requiring interactive communication with the patient/caregiver during the month; however, it is not clear that this code could be utilized for ECG monitoring services through mobile devices. The pre‐existing CPT code 93040 (used for reporting on a Rhythm ECG, 1‐3 leads, without interpretation and report) would not be appropriate for patient initiated mobile device events as this would require an order that is triggered by an event followed by a separate signed and retrievable report. CMS has also proposed establishing a new virtual service HCPCS code, GRAS1, for “Remote Evaluation of Pre‐Recorded Patient Information,” which would reimburse for a provider’s asynchronous review of “recorded video and/or images captured by a patient in order to evaluate the patient’s condition” and determine whether or not an office visit is necessary. This code could be billed separately if there was not an E/M visit within the previous seven days. CMS finalized separate payment for CPT code 99091 (collection and interpretation of physiologic data, e.g., ECG, BP, glucose monitoring) digitally stored and/or transmitted by the patient and/or caregiver to the physician or other qualified HCP, qualified by education, training, licensure/regulation, requiring a minimum of 30 minutes of time. However, there must be a clinically relevant reason for the physician to need to review the data each month.
